# A Quantitative Study of the Hog1 MAPK Response to Fluctuating Osmotic Stress in *Saccharomyces cerevisiae*

**DOI:** 10.1371/journal.pone.0009522

**Published:** 2010-03-04

**Authors:** Zhike Zi, Wolfram Liebermeister, Edda Klipp

**Affiliations:** 1 Centre for Biological Signalling Studies (bioss), University of Freiburg, Freiburg, Germany; 2 Center for Biological Systems Analysis (ZBSA), University of Freiburg, Freiburg, Germany; 3 Humboldt-Universität zu Berlin, Institute of Biology, Theoretical Biophysics, Berlin, Germany; Center for Genomic Regulation, Spain

## Abstract

**Background:**

Yeast cells live in a highly fluctuating environment with respect to temperature, nutrients, and especially osmolarity. The Hog1 mitogen-activated protein kinase (MAPK) pathway is crucial for the adaption of yeast cells to external osmotic changes.

**Methodology/Principal Findings:**

To better understand the osmo-adaption mechanism in the budding yeast *Saccharomyces cerevisiae*, we have developed a mathematical model and quantitatively investigated the Hog1 response to osmotic stress. The model agrees well with various experimental data for the Hog1 response to different types of osmotic changes. Kinetic analyses of the model indicate that budding yeast cells have evolved to protect themselves economically: while they show almost no response to fast pulse-like changes of osmolarity, they respond periodically and are well-adapted to osmotic changes with a certain frequency. To quantify the signal transduction efficiency of the osmo-adaption network, we introduced a measure of the signal response gain, which is defined as the ratio of output change integral to input (signal) change integral. Model simulations indicate that the Hog1 response gain shows bell-shaped response curves with respect to the duration of a single osmotic pulse and to the frequency of periodic square osmotic pulses, while for up-staircase (ramp) osmotic changes, the gain depends on the slope.

**Conclusions/Significance:**

The model analyses suggest that budding yeast cells have selectively evolved to be optimized to some specific types of osmotic changes. In addition, our work implies that the signaling output can be dynamically controlled by fine-tuning the signal input profiles.

## Introduction

**Figure 1:**
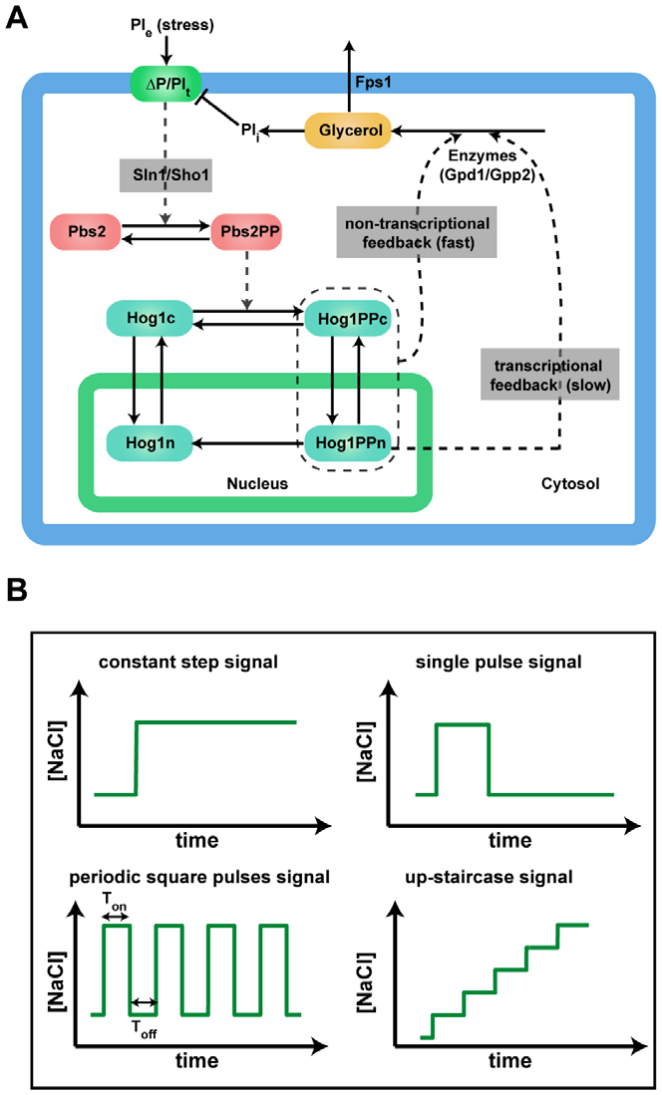
Model scheme for osmosensing network and the osmotic stress signals. (**A**) Scheme of the model for osmosensing network. “Non-transcriptional feedback loop” denotes the Hog1 kinase dependent regulation of glycerol production. “Transcriptional feedback loop” stands for transcriptional regulation of the enzymes responsible for glycerol production. The gray boxes are modeled with coarse-grained black box approaches. (**B**) Different types of osmotic change signals considered in this work.

Cells have evolved to sense and respond to various changes of their environmental conditions such as hormones, nutrients, temperature and osmotic stresses. One of the well-studied examples is the adaption of budding yeast cells (*Saccharomyces cerevisiae*) to high external osmolarity: information about the osmotic stress is transmitted through the high-osmolarity glycerol (HOG) mitogen-activated protein kinase (MAPK) signaling pathway [1]. The increase of external osmolarity is recognized by two osmosensing proteins, Sln1 and Sho1, which in turn independently lead to the phosphorylation of the MAPK kinase (MAPKK) Pbs2. The Sln1 branch first activates two functionally redundant kinases, Ssk2 and Ssk22 (MAPK kinase kinase, MAPKKK), which then activate Pbs2. Upon stress stimulation, the Sho1 branch triggers the activation of a distinct MAPKKK Ste11, which then activates Pbs2. Once the MAPKK Pbs2 is phosphorylated and activated, it subsequently phosphorylates and activates the MAPK Hog1, which shuttles rapidly between the cytoplasm and nucleus. Activated Hog1 accumulates in the nucleus and regulates different processes to increase glycerol accumulation, thereby compensating the increase of external osmolarity [1], [2], [3]. A schematic picture of this process is shown in Figure 1A.

Previous computational and experimental studies have highlighted the response of the key MAPK protein Hog1, especially its phosphorylation and enrichment in the nucleus, to specific profiles of osmotic changes [4], [5], [6], [7], [8], [9], [10], [11], [12]. These analyses revealed the principles of negative feedback for osmo-adaption and demonstrated that Hog1 response is crucial for the regulation of gene expression, cell cycle progression, and osmo-adaption. Moreover, recent studies with microfluidic devices indicated that periodic variations of external osmolarity (input signal) also affect the output of Hog1 response [7], [10], which suggests that the Hog1 MAPK signaling pathway has evolved to selectively respond to different types of osmotic changes. To better understand how budding yeast cells respond to different types of osmotic changes, we developed a mathematical model to investigate the dynamics of the Hog1 response to different scenarios of stress signals: simple step increase, single pulse, periodic square pulses and up-staircase increase of osmotic changes (Figure 1B).

Different modeling strategies have been previously applied to the osmotic stress signaling network to study specific questions. While our previous comprehensive model captured the detailed dynamics and regulation of the osmosensing network [8], [13], other simple models revealed some general properties of the osmo-adaption mechanism of this network [6], [10], [11]. In this work, we keep the model as simple as possible, reducing it to the key components for the regulation of the osmo-adaption process. Our model accounts for the following processes: (1) biophysical changes including internal pressure, external pressure, turgor pressure, and volume changes (for details, see our previous study [8]); (2) The phosphorylation and dephosphorylation of Pbs2 and Hog1; (3) Hog1 nuclear-cytoplasmic shuttling; (4) The regulation of glycerol production and leakage. The scheme of the model is shown in Figure 1A. The development of the model is described in the Methods section.

## Results

### The Mathematical Model Can Reproduce Various Experimental Observations of the Hog1 Response to Osmotic Stress

**Figure 2:**
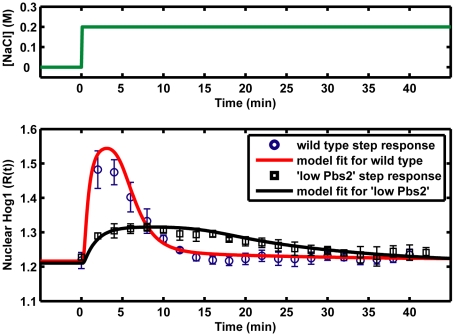
Comparison of model fits to the experimental data sets for step increase of 0.2 M NaCl in wild-type yeast and “low Pbs2” mutant. Circles and squares represent the experimental data sets from Fig. 2D in reference [10]. The solid curves are the simulation result from our model. For the “low Pbs2” mutant, we set Pbs2 concentration in the model to be 12.55% of the corresponding Pbs2 in the “wild type”.

**Figure 3:**
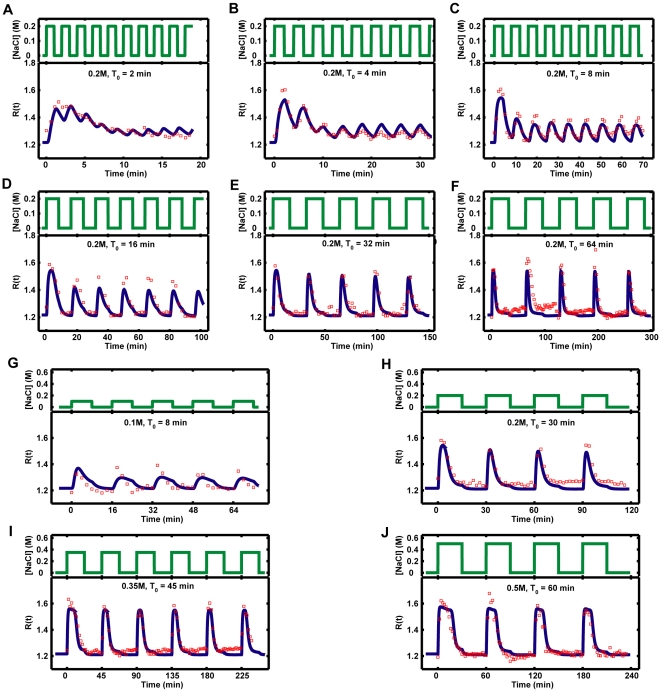
Comparison of model fits to the experimental data sets for periodic square pulses of osmolarity. Red squares represent the experimental data sets from Fig. S2 (**A–F**) and Fig. S5 (**G–J**) in reference [10]. Blue curves are the simulation result from our model in this study. T_0_ is the period of periodic square pulses. In all cases, the time of the pulse in on phase and off phase are equal to half of T_0_ (T_on_ = T_off_ = T_0_/2).

Having established the mathematical model, we needed to estimate the parameter values and to check whether our model was able to reproduce the experimental observed Hog1 response to osmotic stress. We estimated parameter values by fitting them to the experimental data sets generated with micro-fluidic devices. With 22 optimized parameter values, the model fits several hundreds of experimental data points very well. For example, it can reproduce the Hog1 response to a simple step increase of salt stress (NaCl) and its behavior in a Pbs2-underexpressing mutant [10] (Figure 2). In addition, the simulation results quantitatively match the experimental observations of the Hog1 response to different frequencies and strengths of NaCl pulses [10] (Figure 3). The model also confirmed a previous experimental observation that stronger osmotic stress increases the glycerol production rates (Figure S1) [10].

We next challenged our model by testing whether it could predict other observations from previous experimental studies. It is worth noting that these experimental data had not been used for the parameter estimation of the model. First, we checked whether the model could predict the Hog1 response to periodic square pulses of 0.2 M NaCl in a Pbs2 under-expressing strain. Indeed, the model qualitatively reproduced the experimental observations that a reduced expression level of Pbs2 will decrease the maximum response of Hog1 and slows down the adaption of Hog1 response (Figure S2) [10]. In addition, our model predicted that this effect depends on the under-expressing level of Pbs2: Pbs2 should be significantly down-regulated in order to obtain a different Hog1 response profile. Otherwise, the Hog1 responses in “low Pbs2” mutant would resemble the one in the wild type (Figure S3). Furthermore, the model also confirmed our previous results for Hog1 response to a double continuous NaCl stress (Figure S4) [8].

**Figure 4:**
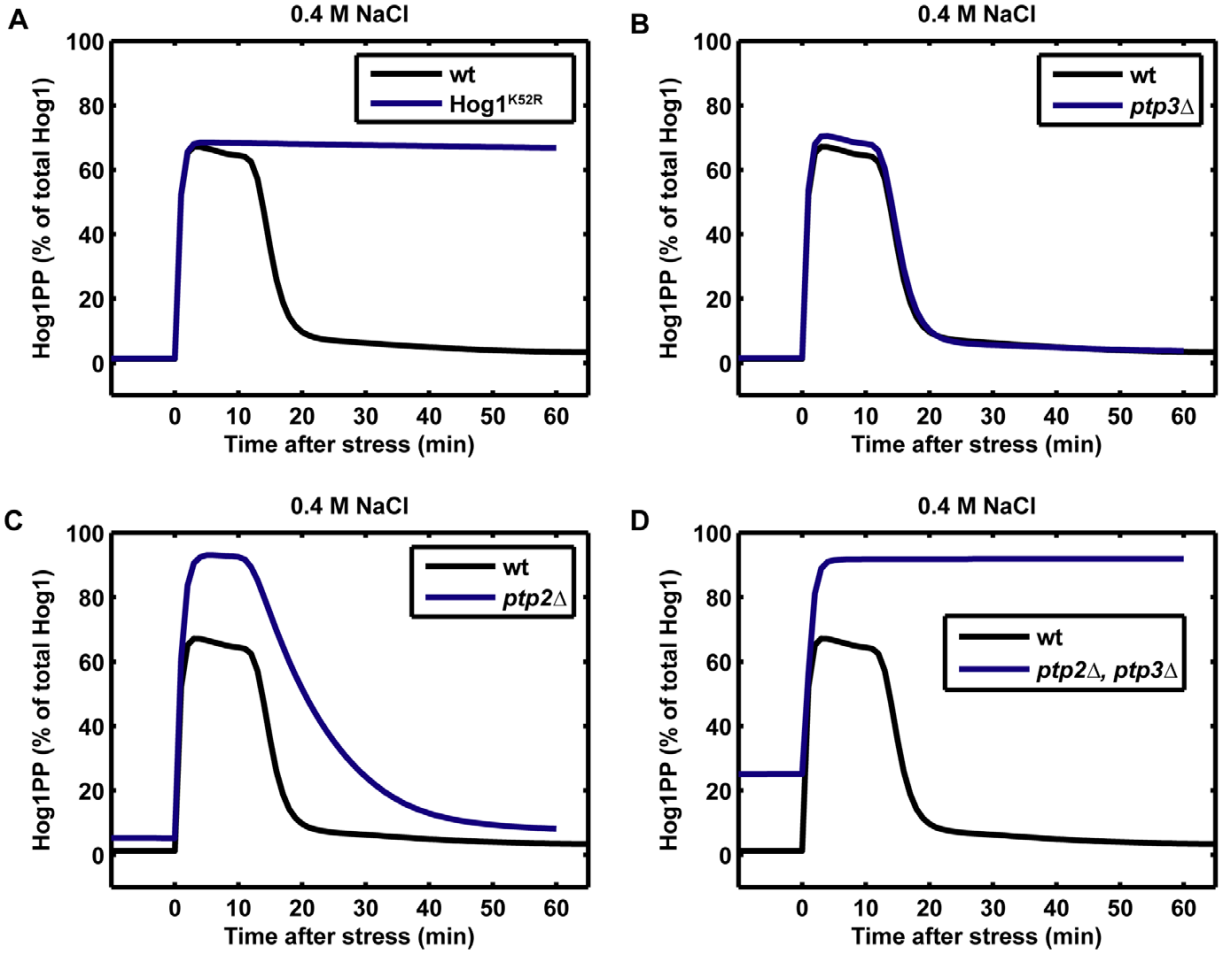
Model predictions for Hog1 phosphorylation response to step increase of 0.4 M NaCl in different mutants. (**A**) Hog1 catalytically inactive mutant (Hog1K52R), In the model, we set ks1_Glyc = 0 for this mutant. (**B**) Knockout of cytoplasmic protein tyrosine phosphatase Ptp3 (*ptp3 Δ*). In the model, we set kdepho_Hog1PPc = 0 for this mutant. (**C**) Knockout of nuclear protein tyrosine phosphatase Ptp2 (*ptp2Δ*). In the model, we set kdepho_Hog1PPn = 0 for this mutant. (**D**) Knockouts of Ptp2 and Ptp3 (*ptp2Δ, ptp3 Δ*). In the model, we set kdepho_Hog1PPc = 0 and Kdepho_Hog1PPn = 0 for this mutant.

Finally, the model agrees well with the experimentally observed Hog1 phosphorylation profiles in different mutants. For example, a mutant with catalytically inactive Hog1 (Hog1^K52R^) shows to a stronger phosphorylation level of Hog1 upon osmotic stress (Figure 4A): this prediction is consistent with previous reported results [6], [9], [14]. We also checked whether the model could correctly predict the effect of different knockout of the protein tyrosine phosphatases, Ptp2 and Ptp3. As shown in Figure 4B–4D, the model confirmed that a knockout of the cytoplasmic protein tyrosine phosphatase Ptp3 (*ptp3Δ*) alone has almost no effect on the Hog1 tyrosine phosphorylation level, while deletion of the nuclear protein tyrosine phosphatase Ptp2 (*ptp2Δ*) or its deletion together with Ptp3 (*ptp2Δ*, *ptp3Δ*) significantly changes the Hog1 phosphorylation profile [15], [16].

### Signal Response Gain: A Measure for Quantifying the Signal Transduction Efficiency

To quantify the signal transduction efficiency of Hog1 response in different scenarios of osmotic stress, we introduced the concept of “signal response gain” (*G_s_*), which is defined as the ratio of the integrated output change to the integrated input (signal) change (Equations 1–3):
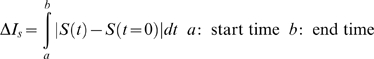
(1)

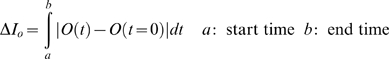
(2)

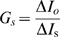
(3)where *S*(*t*) denotes the signal level at time *t* and *S*(*t = *0) denote the basal level of the signal; *O*(*t*) is the output level at time *t* and *O*(*t* = 0) corresponds to the basal level of the output.

**Figure 5:**
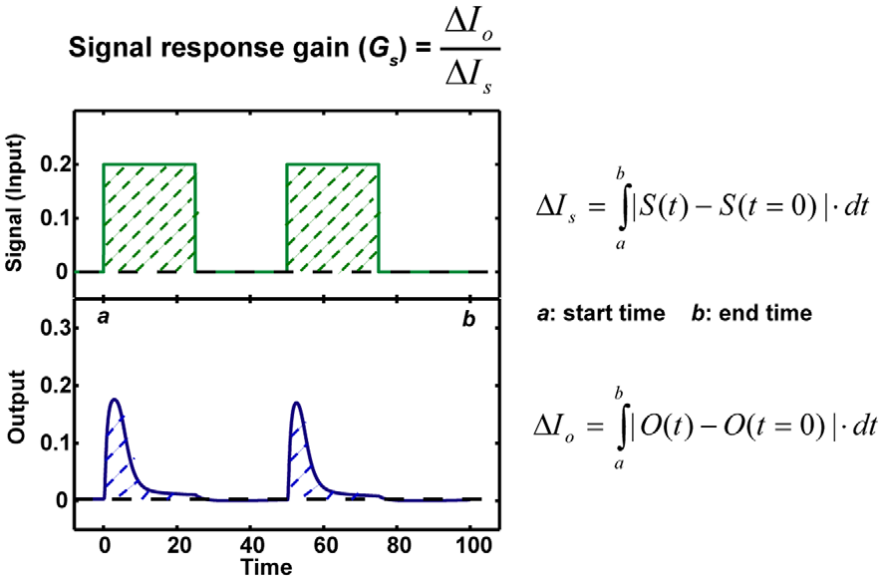
Schematic description of signal response gain (*G_s_*). The integral change of signal input (*ΔI_s_*) corresponds to the area formed by the signal input curve and the basal line of signal input. The integral change of signal output (*ΔI_o_*) corresponds to the area formed between signal output response curve and the basal line of signal output. The signal response gain is defined as the ratio of *ΔI_o_* to *ΔI_s_*.

The signal response gain is schematically illustrated in Figure 5. It represents how much output change is provoked by the input change, both measured with respect to the basal level of the system. Since the signal response gain is normalized to the amount of signal input, it quantifies how efficient the signal output is transmitted from a given amount of signal input. The higher is the value, the more efficient is the signal transmitted from the input to the output. In this study, we choose nuclear phosphorylated Hog1 (denoted as Hog1PPn in the model) as the output of this pathway because it regulates the expression of hundreds of genes (reviewed in [1], [2]). In addition, as nuclear phosphorylated Hog1 (Hog1PPn) is highly correlated with nuclear Hog1 enrichment and the total Hog1 phosphorylation level, its dynamic profiles should be comparable with other experimental observations of nuclear Hog1 localization by imaging analysis [7], [10].

### Hog1 Response to Single Pulse of Osmotic Stress

**Figure 6:**
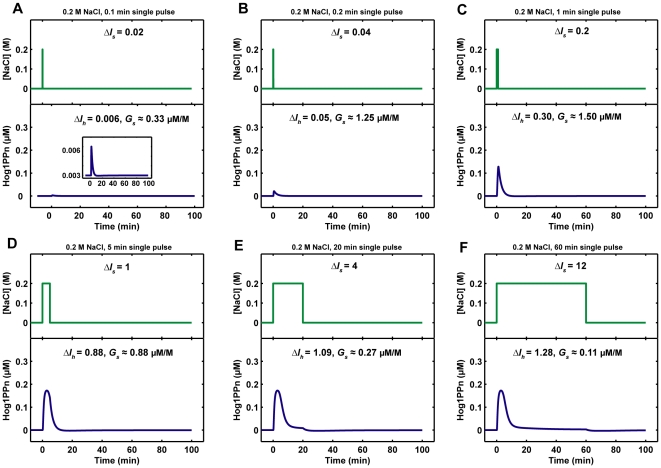
Nuclear phosphorylated Hog1 (Hog1PPn) response to single pulses of 0.2 M NaCl. *ΔI_s_*: NaCl integral change, *ΔI_h_*: integral change of Hog1PPn response, *G_s_*: Hog1PPn response gain. The intersection curve in the lower part of panel A is a zoom-in shown in different scale.

**Figure 7:**
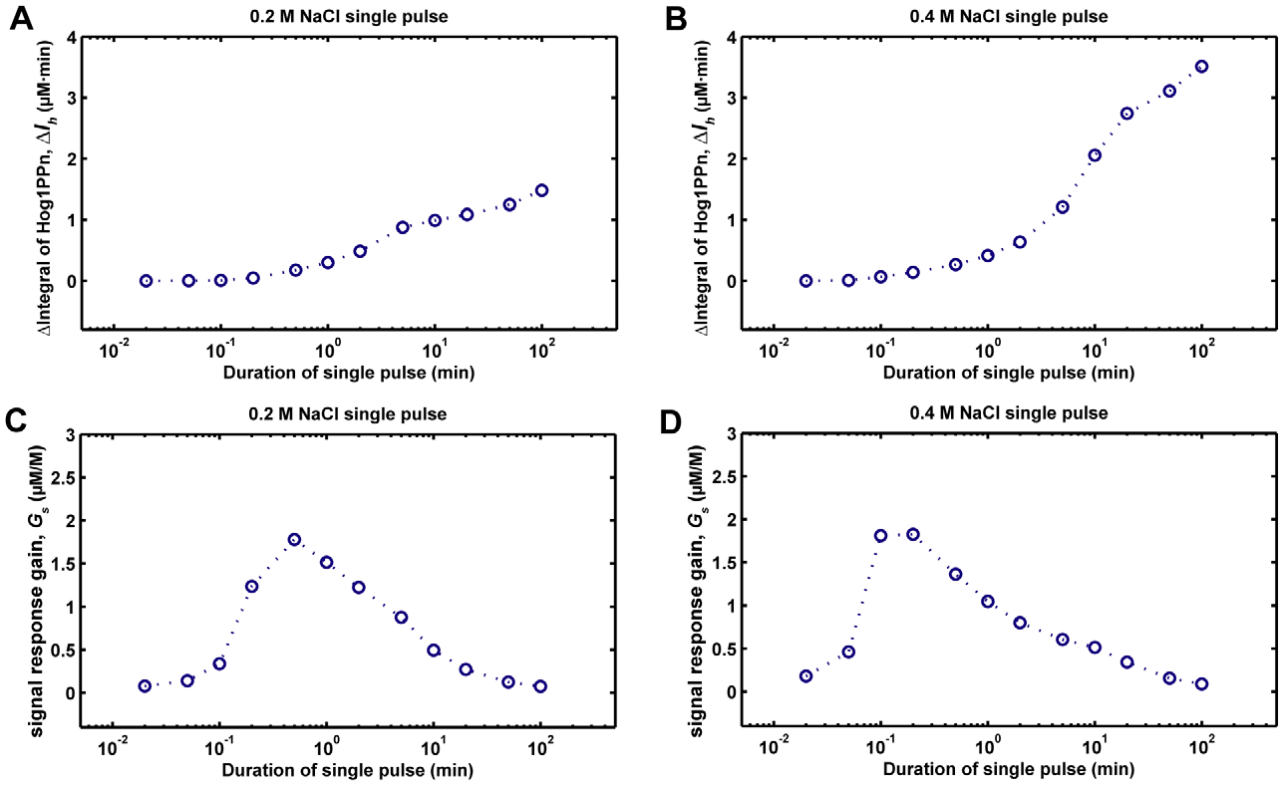
Relationship between nuclear phosphorylated Hog1 (Hog1PPn) response and the duration of single pulses of NaCl change. *ΔI_h_*: integral change of Hog1PPn response. *G_s_*: Hog1PPn response gain.

After calibrating the model by a data-based modeling approach, we used it to study the following question: if cells are exposed to osmotic stress, what is the minimum duration of the stress that is necessary for cells to induce a Hog1 response? To answer this question, we simulated the model with the addition of a single pulse of NaCl. This corresponds to an experiment in which cells are exposed to NaCl stress for a certain period and then switched to fresh medium without NaCl. As shown in Figure 6, when the duration of 0.2 M NaCl single pulse stimulation is too short (< = 0.1 min), there is almost no Hog1 response. However, once the stress stimulation lasts longer than 1 min, Hog1 becomes phosphorylated and its integral level increases with the stimulation time (Figure 7A–7B).

We also quantified the relationship between the Hog1 signal response gain (*G_s_*) and the duration of single pulses of 0.2 M or 0.4 M NaCl, and found that the integral of Hog1 phosphorylation (*ΔI_h_*) grows proportionally to the increase of stress duration (*ΔI_s_*). Interestingly, Hog1 signal response gains (*G_s_*) have bell-shaped response curves with respect to stress durations: as the stress duration grows, the signal response gain first increases, but then decreases again because the cells have already adapted to the osmotic stress after a certain time (Figure 7C–7D). This result implies that efficiency of signal transduction from stress input to integrated Hog1 response is optimized with respect to the stress duration.

### Hog1 Response to Periodic Square Pulses of Osmotic Stress

We next investigated how Hog1 responds to periodic square pulses of osmotic stress, which had been studied before with microfluidic device [7], [10]. Here, the model confirmed previous study results that Hog1 responds periodically to periodic square pulses of NaCl stress at low frequencies. However, at high frequencies, Hog1 did not respond periodically, but showed a similar response as to simple step osmotic stress (Figure S5) [7], [10]. Other simulation results indicate that both the integral change of Hog1 phosphorylation (*ΔI_h_*) and Hog1 response gain (*G_s_*) have bell-shaped response curves with respect to the frequency of stress pulses (Figure S6). Similar as the model analysis for Hog1 response to a single pulse of stress change, the signal transduction efficiency for the integral of the Hog1 phosphorylation is also optimized to a frequency of periodic stress change (with period of about 1 min).

**Figure 8:**
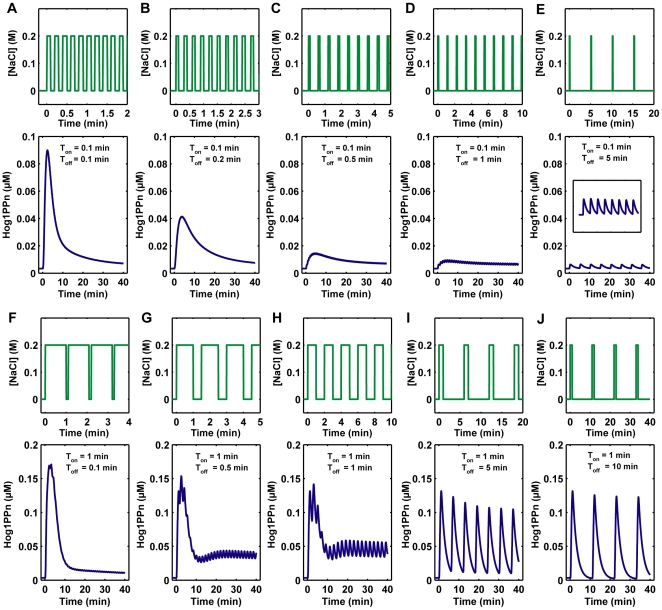
Nuclear phosphorylated Hog1 (Hog1PPn) response to different periodic square pulses of 0.2 M NaCl. (**A–E**) The time of pulse in on phase (T_on_ = 0.1 min) of periodic square pulses is very short, while the time of pulse in off phase (T_off_) of periodic square pulses varies from 0.1 min to 5 min. The intersection curve in low part of panel E is a zoom-in of Hog1PPn response in different scale. (**F–J**) The time of periodic square pulse in on phase (T_on_ = 1 min) is long enough to induce Hog1PPn response, the time of periodic square pulse in off phase (T_off_) varies from 0.1 to 10 min.

We compared the model simulations for Hog1 response to single pulses and periodic square pulses of osmotic stress. It is interesting that Hog1 almost does not respond to a short single pulse of 0.2 M NaCl with a duration of 0.1 min (Figure 6A), but it responds to periodic square pulses of 0.2 M NaCl with period of 0.2 min (T_0_ = 0.2 min, Figure S5A). So why can cells gain a Hog1 response from periodic NaCl stress at a high frequency, but do not respond to a single pulse of NaCl that is removed very quickly (0.1 min) after the first stimulation? From our model simulations for a single pulse of 0.2 M NaCl, we know that the necessary activation time for a Hog1 response is about 1 min. This suggests the following explanation: cells will respond if they are exposed to sufficient osmotic stress during the first minute, no matter whether it is a single step stimulation or several pulses. Otherwise, cells will not respond if they are not sufficiently stimulated within 1 minute. We tested this hypothesis by adding different NaCl stimulations as inputs to the model and monitored the corresponding Hog1 response. First, 0.2 M NaCl is added for 0.1 min (time of pulses in on phase, T_on_ = 0.1 min), then it is removed for different duration and 0.2 M NaCl are subsequently added again. The time gap between two continuous 0.2 M NaCl pulses is defined as the time of pulses in off phase (T_off_), which was also used in a previous study [7]. As shown in Figure 8A–8E, when the pulse length is very short (T_on_ = 0.1 min) and if the second subsequent 0.2 M NaCl pulse does not come within 1 min (T_off_ ≧ 1 min), the maximum amplitudes of Hog1 responses are very weak. However, if the first stress stimulation is sufficient (T_on_ ≧ 1 min), then the off time of the pulses (T_off_) does not have a significant effect on the maximum amplitude of the Hog1 response (Figure 8F–8J and data not shown). These simulation results also confirmed a previous experimental observation that if the off time of pulses (T_off_) is too short, cells cannot distinguish different pulses and respond as if they are exposed to a constant osmotic stress [7].

### Hog1 Response to an Up-Staircase/Ramp Input of Osmotic Stress

**Figure 9:**
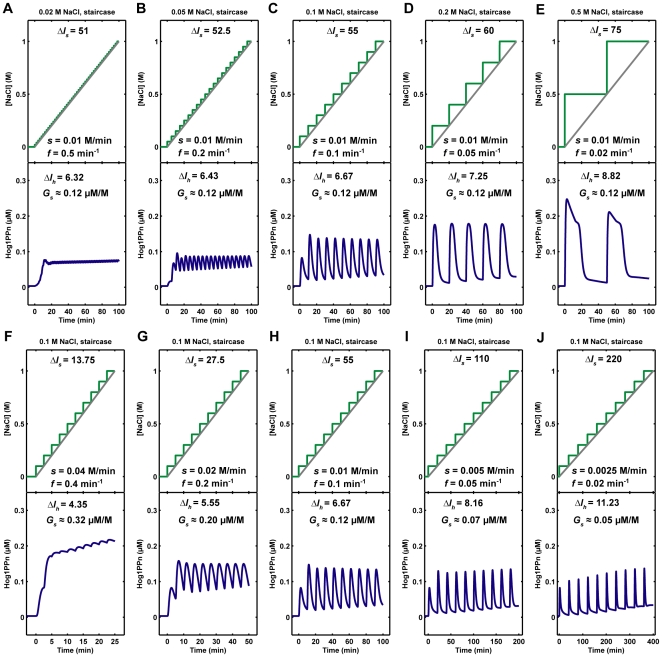
Nuclear phosphorylated Hog1 (Hog1PPn) response to different staircase NaCl stimulations. *ΔI_s_*: NaCl integral change, *ΔI_h_*: integral change of Hog1PPn response, *G_s_*: Hog1PPn response gain, *s*: slope of the staircase profile of NaCl, which is the ratio of final NaCl concentration to the duration of NaCl stimulation. *f*: frequency of the staircase steps, which corresponds to the inverse of the interval between staircase steps.

Recently, during the preparation of this manuscript, Muzzey et al. have shown that Hog1 cannot perfectly adapt to a ramp input of osmotic stress [11]. Although this result was not taken into account during the construction of our model, the conclusion was independently verified by our model simulations (Figure S7). We implemented more analyses of the relationship between Hog1 response and the profiles of different up-staircase/ramp inputs of osmotic stress. The simulation results showed that Hog1 can adapt well, although not perfectly, to an up-staircase input of osmotic stress when the frequency of up-staircase steps (the inverse of duration of each staircase step) is below a certain threshold (*f*<0.1 min^−1^, Figure 9D, 9E, 9I, 9J). It is worth noting that the adaption of Hog1 response in these cases is not perfect because after each step of osmotic increase, Hog1 activity reaches a slightly higher level than before. On the other hand, when up-staircase stress changes are too fast (*f* >0.2 min^−1^, which resembles a ramp input of stress), Hog1 cannot adapt and will persist at a high response level (Figure 9A, 9F).

In addition, the model simulations indicate that the integral of Hog1 response (*ΔI_h_*) grows with the integral of the osmotic stress input (*ΔI_s_*) (Figure 9). More interestingly, the Hog1 response gain (*G_s_*, ratio of *ΔI_h_* to *ΔI_s_*) depends on the slope of the up-staircase. If different up-staircase profiles have the same slope, then Hog1 response gains are similar and do not depend on the frequency of up-staircase steps (Figure 9A–9E). However, the Hog1 response gain declines with the reduction of up-staircase slope (Figure 9F–9J). Taken together, our model suggests that the step frequency in up-staircase signal affects the adaption performance of Hog1 response, while the slope of the up-staircase signal is the key factor that determines the overall Hog1 response gain.

## Discussion

### Relevance of the Model to Different Experimental Setups

The model presented in this work was calibrated with data sets of Hog1 nuclear enrichment in budding yeast cells upon changing salt concentration, measured with the microfluidic device [10]. In this experimental setup, cells are exposed to a constant flow of external media which come from different reservoirs [7], [10]. The external concentration of glycerol is kept constant at a level close to zero. In contrast, in a normal Erlenmeyer flask system, cells are exposed to a medium with high osmolarity, in which the external glycerol level is also increased. Therefore, different experimental devices might cause some quantitative difference of the Hog1 response. In addition, different kinds of high-osmolarity or different budding yeast strains also affect the quantitative dynamics of Hog1 response. Nevertheless, the qualitative dynamics of Hog1 responses in different experimental setups should be similar and consistent. When we compare the model predictions with other experimental data obtained in different experimental setups, we need to focus on the qualitative principles of Hog1 response.

### Comparison to the Concise Linear Time Invariant Model

Mettetal *et al.* developed a concise linear time invariant (LTI) model for predicting the Hog1 response to periodic square pulses of osmotic changes [10]. Although the LTI model has only two differential equations describing three major feedbacks, it revealed that the dynamics of the osmo-adaptation response are mainly controlled by the fast Hog1-dependent negative feedback loop and suggested that changes in gene expression have a minor effect on Hog1 response. The predictions from this simple model were later confirmed by experimental analysis and our new model analyses. However, a detailed comparison of the LTI model prediction to the experimental data sets shows that with 4 parameters, this model is too simple to fully reproduce the quantitative dynamics of the Hog1 nuclear enrichment (Figure S8, Figure S9). Not surprisingly, due to more number of the parameters, our new model fits the time course dynamics of Hog1 response better than the LTI model (Figure S8–S9). In particular, the experimental data sets and our model simulations suggest that budding yeast cells can remember the first pulse of high osmolarity and need less time to adapt to the subsequent pulses of stimulation (Figure S9G–S9H). The LTI model fails to capture this dynamic property because the integral feedback property of glycerol accumulation was not modeled in this simple model (Figure S9C–S9D). Muzzey *et al.* from the same group later found that Hog1-dependent glycerol accumulation is crucial for the perfect adaption of budding yeast to simple step increase of osmotic change and they also proposed a revised concise model taking glycerol production into account [11]. This shows that the regulation of glycerol accumulation is important for controlling the dynamics of both Hog1 response and osmo-adaption in budding yeast.

### Hog1 Phosphorylation and Nuclear Localization Are Highly Correlated in Different Scenarios of Osmotic Stresses

Previous experimental studies have shown that there is a correlation between Hog1 phosphorylation and its nuclear localization upon constant osmotic stress stimulation [17], [18], [19]. Accordingly, Hog1 nuclear enrichment has been used as an indicator for the Hog1 response to normal and periodic osmotic stresses because it is convenient for imaging analysis [7], [10], [11], [17], [18], [19]. Here, we took advantage of mathematical analysis to check the correlation between Hog1 phosphorylation and nuclear localization upon different kinds of osmotic stress: simple step increase, periodic square pulses and up-staircase increase of osmotic changes. The simulation results indicate that total phosphorylated Hog1 (x_1_), nuclear Hog1 (x_2_) and nuclear phosphorylated Hog1 (x_3_) are highly correlated in all three stress stimulation scenarios (Figure S10). The correlation coefficients between each pair of these three variables (x_1_, x_2_, x_3_) are larger than 0.98, with p-values of almost 0, which means that their correlations are highly significant. Therefore, our model analysis quantitatively supports the assumption that Hog1 nuclear enrichment is a good indicator for studying Hog1 response upon osmotic stress, which had been extensively used before.

### Hog1 Dependent Non-Transcriptional Regulation of Glycerol Production Is the Key Factor Controlling Perfect Osmo-Adaption

**Figure 10:**
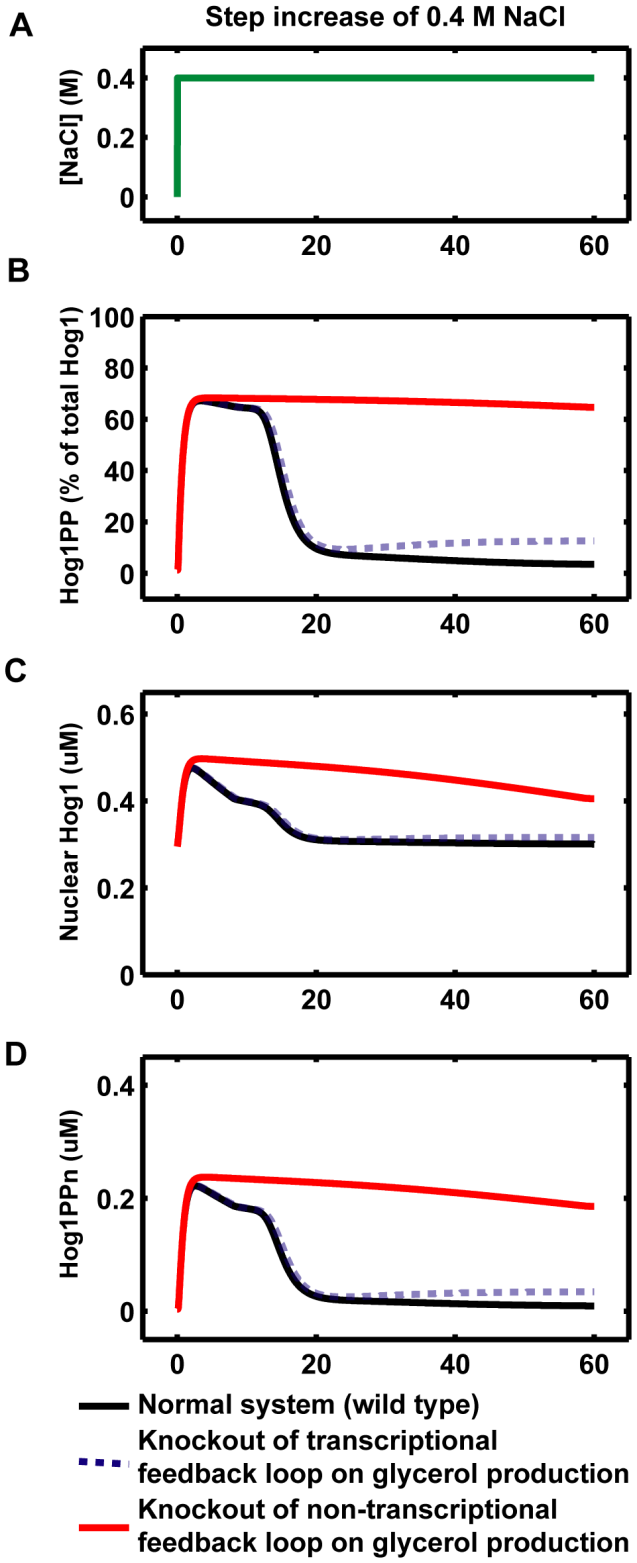
Model predictions of Hog1 responses for the knockouts of different feedback loops involved in glycerol production. We set 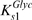 = 0 to simulate the knockout of the non-transcriptional feedback loop (Hog1 kinase, totalHog1PP, dependent) on glycerol production. The simulation results are shown in red curves in panel B–D. We set 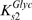 = 0 to simulate the knockout of the transcriptional feedback loop (nuclear phosphorylated Hog1, Hog1PPn, dependent) on glycerol production. The simulation results are shown in blue dotted curves in panel B–D.

Previous studies implied that the transcriptional feedback loop on glycerol production (slow feedback loop denoted in Figure 1A) plays only a minor role in the regulation of osmo-adaption [3], [10]. To evaluate different contributions of the transcriptional and non-transcriptional feedbacks on the osmo-adaption ability, we implemented *in silico* knockouts of the fast non-transcriptional and the slow transcriptional feedback loops on glycerol production, respectively. As shown in Figure 10, the simulations suggest that the perfect adaption of Hog1 is only slightly affected by the knockout of the slow transcriptional feedback loop. However, perfect adaption is almost lost when the non-transcriptional feedback loop (Hog1 kinase dependent) is knocked out (Figure 10). Therefore, our model independently confirmed the recent experimental result that perfect adaptation requires Hog1 kinase activity, which regulates glycerol production [11].

## Methods

### Model Development

#### (1) Model assumptions

We made the following assumptions during the development of this model.

(1) Although cell growth is observed after cell adaption to osmotic stress, we ignore the effect of cell growth on the volume change during the time scale of osmo-adaption.

(2) When cells are exposed to constant osmotic stress, their volumes decrease and then reach the initial volume again. Previous studies indicate that the nuclear volume of yeast cells also changes during growth [20]. Here we assume that the volumes of cytoplasm and nucleus change simultaneously and that their ratio does not change. In other words, the cytoplasmic volume and nuclear volume both change proportionally to their initial volume percentage of the whole cell volume.

(3) Before cells are exposed to osmotic stress, we assume that the system is in a steady state, which means that the Hog1 distribution in cytoplasm and nucleus, the glycerol and the Pbs2 phosphorylation level are constant before osmotic stress stimulation.

#### (2) Modeling of Pbs2 activation

Our previous integrative model [8] contains a phospho-relay system, in which the SLN1 and SHO1 branches sense the osmotic stress signal and both activate the MAPKK, Pbs2. Here, we simplified and modeled the phosphorylation of Pbs2 with a Hill function using turgor pressure as input (Equation 4). During parameter estimation, we found that a large value of the Hill coefficient (with a value of 8 in this model) is necessary for fitting the experimental data sets well, which suggests a non-linear cooperative effect for Pbs2 phosphorylation in the phospho-relay system. The rate of Pbs2 phosphorylation reaction is
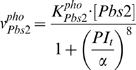
(4)


#### (3) Modeling of glycerol production

Moreover, we simplified the metabolic regulation process for the production of glycerol and considered two regulation levels of glycerol production by Hog1: (1) non-transcriptional feedback on the enzyme activities and (2) transcriptional feedback on the synthesis of the enzymes that regulate glycerol production. Westfall *et al.* have shown that the non-transcriptional feedback is independent on Hog1 nuclear localization [3]. Here, we use a Hill function to model the non-transcriptional feedback on glycerol synthesis, with a total Hog1 phosphorylation level that reflects the overall Hog1 kinase activity (Equation 5).
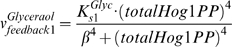
(5)where 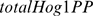 denotes the total percentage of Hog1 molecules being phosphorylated.

In the transcriptional feedback loop on glycerol production, active Hog1 induces the expression of the glycerol-producing enzymesGpd1 and Gpp2. This process involves gene expression and protein translation. Therefore, there is a time delay between the activated nuclear Hog1 and the production of Gpd1 and Gpp2. We used a linear chain of length *N* variables with linear differential equations to model the delay between nuclear Hog1 activity and the transcriptional feedback on the expression of glycerol-producing proteins, which are symbolized as *Yt* in the model [21].
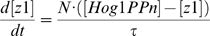
(6)

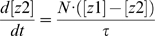
(7)

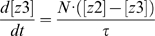
(8)

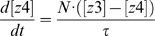
(9)

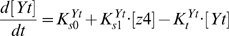
(10)

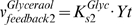
(11)


We set *N* = 4, 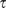 (delay time) = 20 min. This represents a reasonable approximation of the time delay for the production of glycerol-producing proteins *Yt* after an osmotic shock [8], [10].

We also consider a constitutive Hog1-independent production of glycerol and model it with the following rate equations.
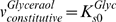
(12)


#### (4) Modeling of glycerol leakage

Previous studies indicated that the glycerol channel protein Fps1 might contribute to the regulation of intracellular glycerol accumulation [22]. Similar to our earlier comprehensive model, we modeled the effect of the Fps1 glycerol channel on glycerol leakage with a switch-like Hill function linked to turgor pressure (*PI_t_*). With equation (12), the model assumes that the Fps1 glycerol channel closes when the turgor pressure disappears in response to high osmolarity stress.
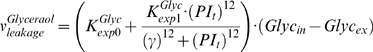
(13)where 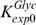 is the diffusion constant for Fps1-independent export of glycerol and 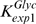 corresponds to the maximum export rate of glycerol through the Fps1 channel. *Glyc_in_* denotes the intracellular glycerol concentration; *Glyc_ex_* stands for extracellular glycerol concentration, which is assumed to be a constant with a value of 0 in the flow cell microfluidic system.

#### (5) Kinetics for other reactions

All other reactions included in this model are modeled by mass-action kinetics. We did not choose Michaelis-Menten kinetics for these signaling transduction steps because this would require that the total concentration of the enzyme (also being a substrate in signaling pathways) concentration is much smaller than the substrate concentration, which may not be valid. Nevertheless, we tried Michaelis-Menten kinetics for some of these reactions. This increases the number of parameters but did not improve the fit to the experimental data sets.

### Model Availability

The initial conditions, parameter values, and the whole system of ordinary differential equations are provided in the Tables S1, Table S2, Table S3. As this model includes volume changes and discrete events describing stress signal change, most current systems biology markup language (SBML) supporting tools cannot deal with these situations [23]. The Matlab source code for the model and simulations is provided in Code S1. The simulations in this study were done with a revised version of our customized program SBML-SAT [24] in Matlab and they are consistent with those solved with Matlab ODE solver.

### Parameter Estimation

#### (1) Osmotic pressures

For the osmotic pressure values in the unstressed cells, we used the values from our previous integrative model [8], namely 0.875×10^6^ J m^−3^ for turgor pressure (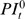), 1.5×10^6^ J m^−3^ for internal osmotic pressure (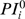) and 0.625×10^6^ J m^−3^ for external osmotic pressure (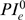) for unstressed cells.

#### (2) Cell volume size

The reported cell volume values of yeast cells are estimated to lie in the range of 37–83 fL [25], [26], [27]. Biswas *et al.* estimated that the cytosol and nucleus occupy about 50% and 7% of the total cell volume in yeast cells of *Exophiala dermatitidis*, respectively [28]. The simulations of our model are not very sensitive to the percentage of cytosol and nucleus. Based on this information, we set the cell volume size (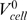) to be 58 fL. The cytoplasmic volume (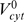) is 29 fL and nuclear volume (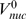) is 4.06 fL. The fixed part of the cell volume (*V_b_*), which is not affected by osmotic changes, is set as 40% of the total volume, corresponding to 23.2 fL. The osmotic volume before osmotic change (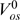) is 34.8 fL. We modeled osmotic volume change by equation (13), similar to our previous model [8].
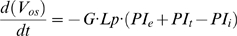
(14)


#### (3) Estimation of the unknown parameter values

Although we chose some parameter values based on the reported values in the literature, there are still 22 parameter values which were not known and should be estimated. Here we used a global optimization algorithm (SRES) to minimize the sum of squared differences between experimental data sets and the model simulation results. SRES (stochastic ranking evolution strategy) is an evolutionary optimization algorithm, which is encoded in our software SBML-PET [29]. SBML-PET is able to simultaneously fit the data sets generated from NaCl stress pulses of different frequencies. We estimated 22 unknown parameters values of the model by fitting to several hundreds of data points generated from 13 different conditions of NaCl stimulations [10]. We used these data sets from Mettetal *et al.* for parameter estimation: the data sets for constant 0.2 M NaCl stimulation (Fig. 2D in reference [10]), 0.2 M NaCl pulse stimulation at different frequencies (Fig. S2 in reference [10]) and different strength of NaCl pulse stimulation (Fig. S5 without CHX in reference [10]). The raw data in the studies of Mettetal *et al.* were quantified by the Hog1 nuclear localization function *Rt*, which is defined as:
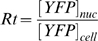
(15)


In our model, the *Rt* value is correspondingly defined as:
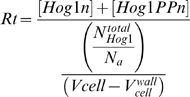
(16)where 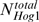, the total number of Hog1 molecules per cell, is set with value of 6780 according to previously reported values [30]. 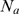 = 6.02×10^23^ is the Avogadro's number. 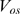 is the osmotic volume and 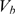 is the fixed part of the cell volume that is not affected by osmotic stress (for details, see our previous model [8]). YFP proteins are not expressed in the cell wall (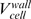), which occupies about 20% percentage of the whole cell volume [28]. Therefore, the whole cell concentration defined in Mettetal *et al.* study should be redefined by subtracting the cell wall volume in our model.

#### (4) Initial conditions

We assume that the system starts in a steady state. In order to estimate the initial concentration of the proteins in the model, we first estimated the total number of molecules per cell for the proteins, using the yeast GFP Fusion Localization Database (http://yeastgfp.ucsf.edu) [30]. Since the initial conditions of the components in the system are given by the steady state values and therefore depend on the other kinetic parameter values, we updated the initial conditions of the proteins during the parameter estimation and forced the system to be approximately in steady state. In the end, the total number of proteins per cell matched the value reported in the yeast GFP database [30]. The values for the initial conditions are summarized in Table S1.

## Supporting Information

Figure S1 Comparison of model fit to the data of glycerol change under square pulse 0.5 M NaCl (T_on_ = T_off_ = 30 min). Change in total glycerol levels over 20 min was used to measure the rate of glycerol production. Experimental data points are plotted by scaling to the model prediction results. Experimental data source: Fig. S6C of the reference (Mettetal *et al.*, Science, 2008, 319: 482–484)
(0.27 MB TIF)

Figure S2 Comparison of model predictions to experimental data of Hog1 nuclear enrichment under periodic square pulse of 0.2 M NaCl. Blue curves: model prediction results. Red square points: experimental data from Fig. S4 of the reference (Mettetal *et al.*, Science, 2008, 319: 482–484). For “low Pbs2” mutant, the model set Pbs2 concentration to be 12.55% of the corresponding Pbs2 in “wild-type”.
(0.39 MB TIF)

Figure S3 Model predictions of Hog1 phosphorylation response to step increase of 0.2 M NaCl in different low expression level of Pbs2 mutants.
(0.30 MB TIF)

Figure S4 Model predictions for Hog1 phosphorylation response to different double step increases of 0.5 M NaCl. (A) Double stress profile. (B–D) Black curves corresponding to Hog1 phosphorylation response to normal step increase of 0.5 M NaCl (single stress). Blue curves are the model predictions for Hog1 phosphorylation response to double stresses (The second stress added at different times). The Hog1 phosphorylation level is normalized to its maximum level in single stress.
(0.35 MB TIF)

Figure S5 Model predictions for nuclear phosphorylated Hog1 (Hog1PPn) response to different periodic square pulses of 0.2 M NaCl. In all cases, the on time (T_on_) and off time (T_off_) of square pulses are half of the period time (T_0_): T_on_ = T_off_ = T_0_/2. *ΔI_s_*: NaCl integral change, *ΔI_h_*: integral change of Hog1PPn response, *G_s_*: Hog1PPn response gain.
(0.50 MB TIF)

Figure S6 Relationship between nuclear phosphorylated Hog1 (Hog1PPn) response and the duration of periodic square pulses of NaCl change. *ΔI_h_*: integral change of Hog1PPn response. *G_s_*: Hog1PPn response gain.
(0.33 MB TIF)

Figure S7 Model prediction for nuclear phosphorylated Hog1 (Hog1PPn) response to ramp increase of NaCl.
(0.24 MB TIF)

Figure S8 Comparison of the data fitting of the LIT concise model and our new model for periodic square pulses of 0.2 M NaCl. The red circle points are the experimental data sets in Fig. S2 of the reference (Mettetal *et al*., Science, 2008, 319: 482–484).
(1.01 MB TIF)

Figure S9 Comparison of the data fitting of the LIT concise model and our new model for different periodic square pulses of NaCl stimulation. The red circle points are the experimental data sets in Fig. S5 of the reference (Mettetal *et al*., Science, 2008, 319: 482–484).
(0.97 MB TIF)

Figure S10 The total amount of phosphorylated Hog1 (x_1_, Hog1PP, quantified as % of total Hog1), nuclear Hog1 enrichment (x_2_, nuclear Hog1) and nuclear phosphorylated Hog1 (x_3_, Hog1PPn) are highly correlated under different types of NaCl stimulation. Numbers in the squares of the correlation map (A, F, K) denote the correlation coefficients of two variables. Numbers within parentheses correspond to the rounded p-values for testing the hypothesis of uncorrelated variables. If the p-value is small (<0.05), then the correlation of the two variables is significant. The correlation coefficient and p-values were calculated with the “corrcoef” function in Matlab. (A–E) Simple step increase of NaCl. (F–J) Periodic square pulses of NaCl. (K–O) Up-staircase increase of NaCl.
(0.76 MB TIF)

Table S1 Initial conditions of the state variables.
(0.05 MB DOC)

Table S2 Complete list of model parameter values.
(0.10 MB DOC)

Table S3 Complete list of ordinary differential equations and other equations.
(0.10 MB DOC)

Code S1 Matlab code for some of the model simulations.
(0.00 MB ZIP)
