# Supplementary figures and images for: A Quantitative Study of the Hog1 MAPK Response to Fluctuating Osmotic Stress in Saccharomyces cerevisiae
Source: PLoS One. 2010 Mar 4;5(3):e9522. doi: 10.1371/journal.pone.0009522 (PMC2831999; doi:10.1371/journal.pone.0009522)

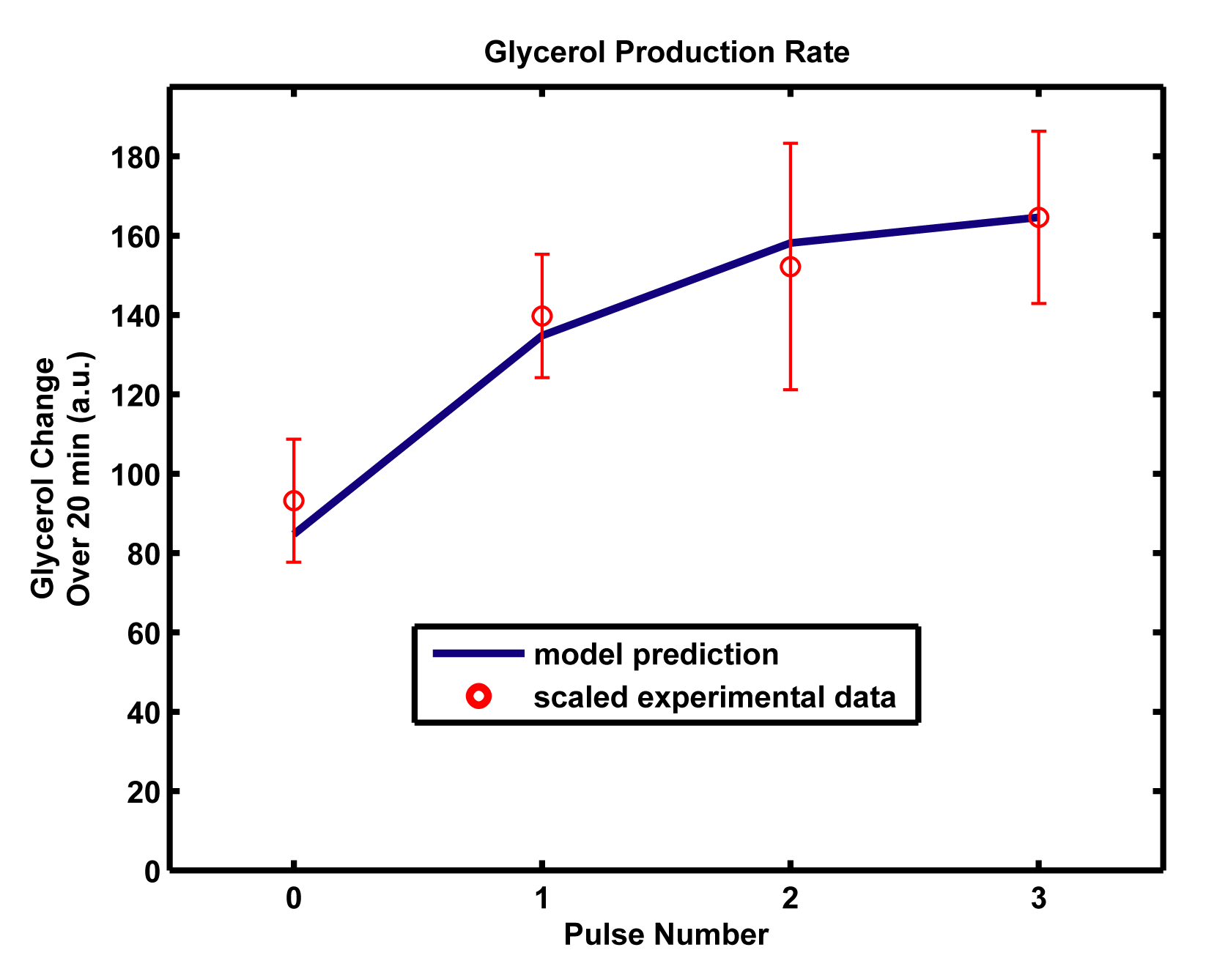

Supplement: Figure S1 — Comparison of model fit to the data of glycerol change under square pulse 0.5 M NaCl (Ton = Toff = 30 min). Change in total glycerol levels over 20 min was used to measure the rate of glycerol production. Experimental data points are plotted by scaling to the model prediction results. Experimental data source: Fig. S6C of the reference (Mettetal et al., Science, 2008, 319: 482–484) (0.27 MB TIF) [file pone.0009522.s001.tif]

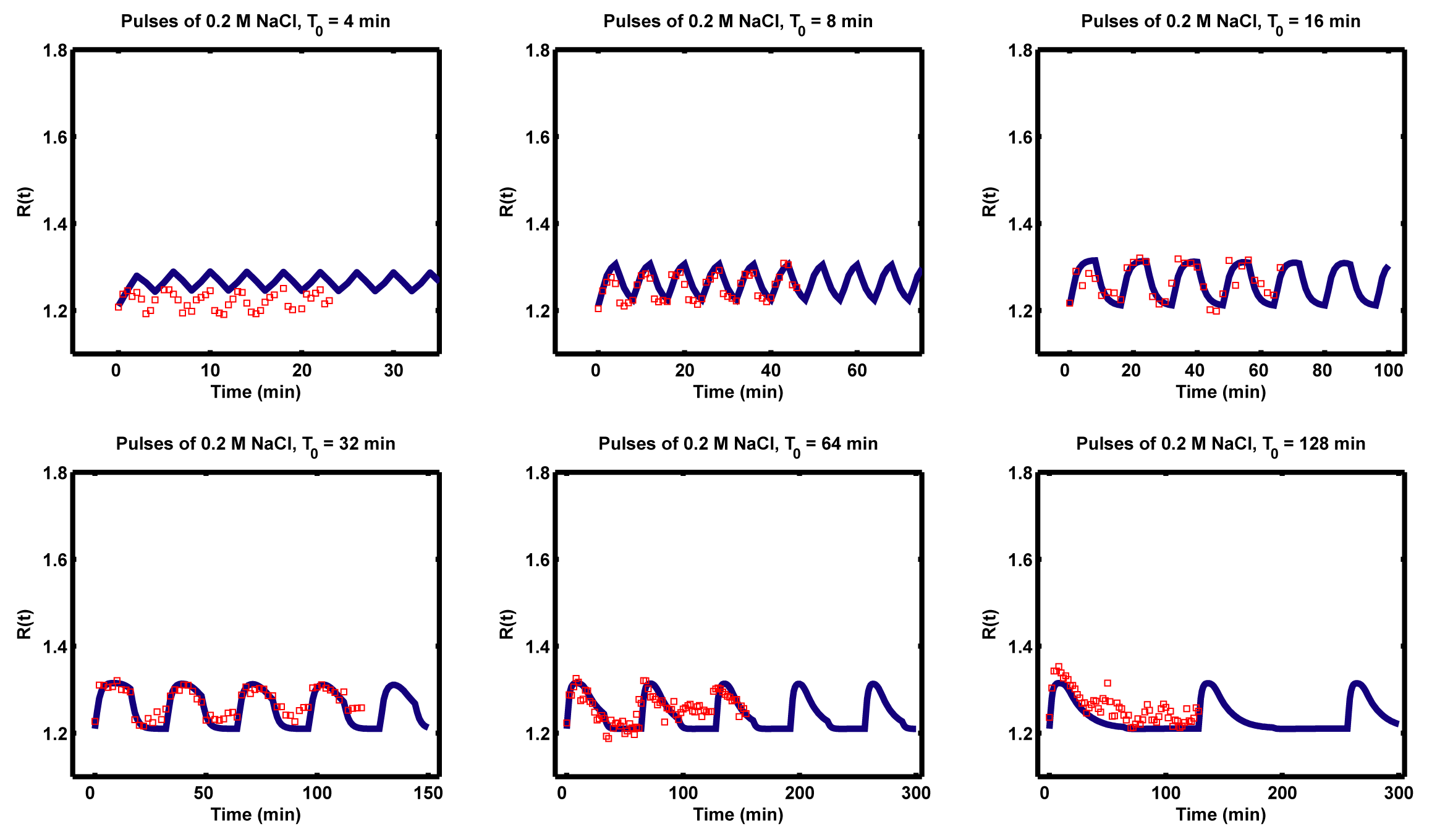

Supplement: Figure S2 — Comparison of model predictions to experimental data of Hog1 nuclear enrichment under periodic square pulse of 0.2 M NaCl. Blue curves: model prediction results. Red square points: experimental data from Fig. S4 of the reference (Mettetal et al., Science, 2008, 319: 482–484). For “low Pbs2” mutant, the model set Pbs2 concentration to be 12.55% of the corresponding Pbs2 in “wild-type”. (0.39 MB TIF) [file pone.0009522.s002.tif]

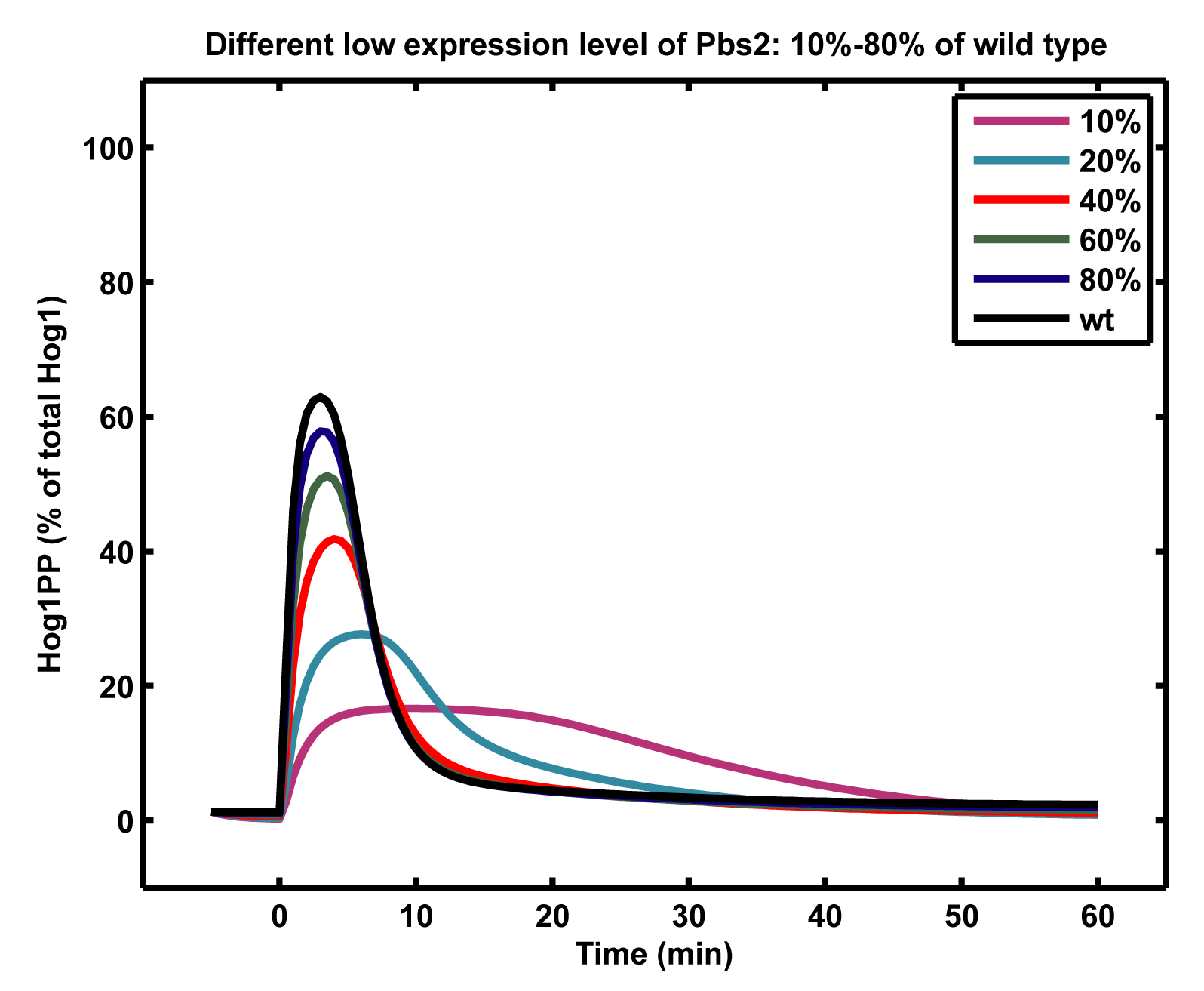

Supplement: Figure S3 — Model predictions of Hog1 phosphorylation response to step increase of 0.2 M NaCl in different low expression level of Pbs2 mutants. (0.30 MB TIF) [file pone.0009522.s003.tif]

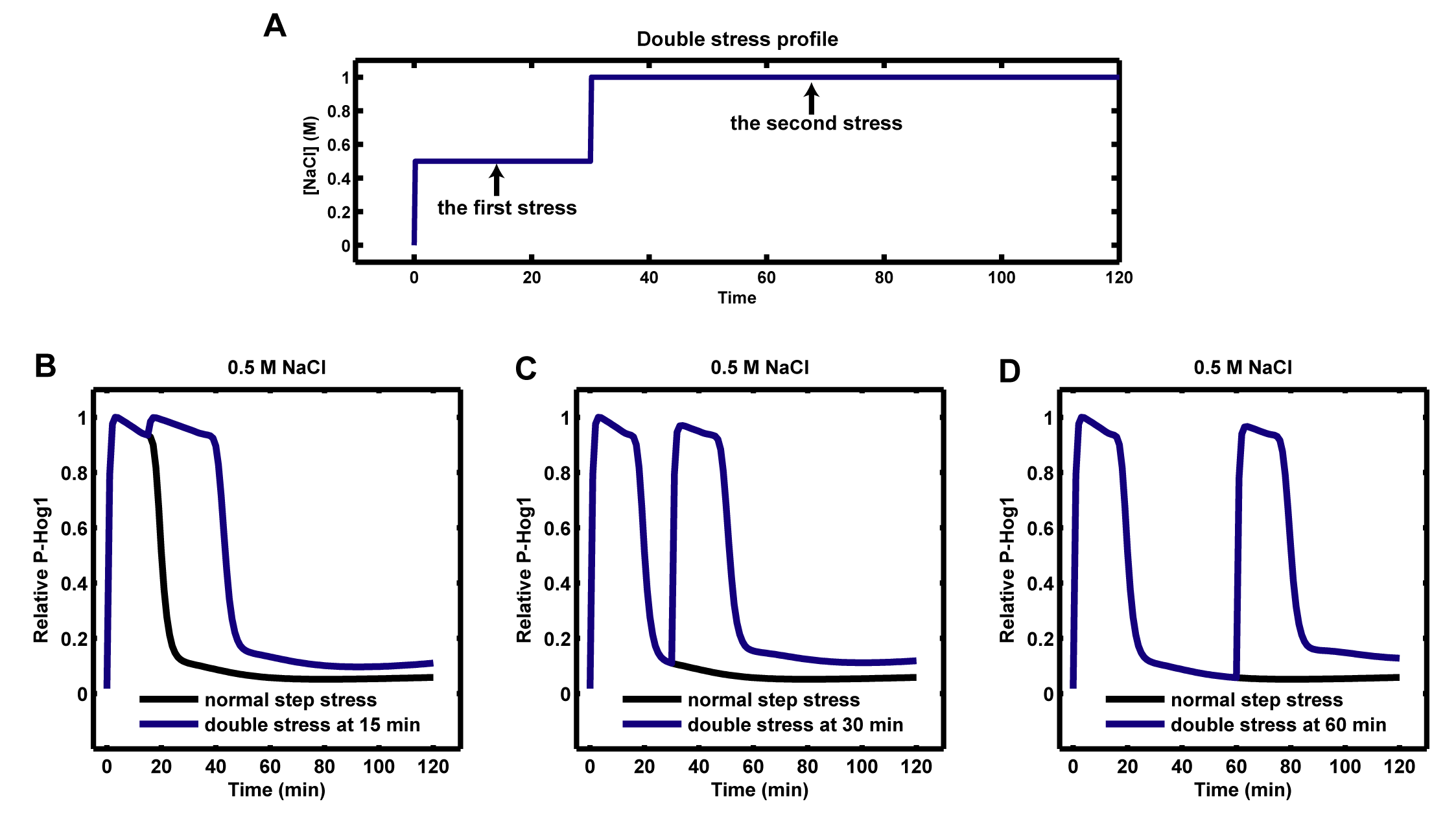

Supplement: Figure S4 — Model predictions for Hog1 phosphorylation response to different double step increases of 0.5 M NaCl. (A) Double stress profile. (B–D) Black curves corresponding to Hog1 phosphorylation response to normal step increase of 0.5 M NaCl (single stress). Blue curves are the model predictions for Hog1 phosphorylation response to double stresses (The second stress added at different times). The Hog1 phosphorylation level is normalized to its maximum level in single stress. (0.35 MB TIF) [file pone.0009522.s004.tif]

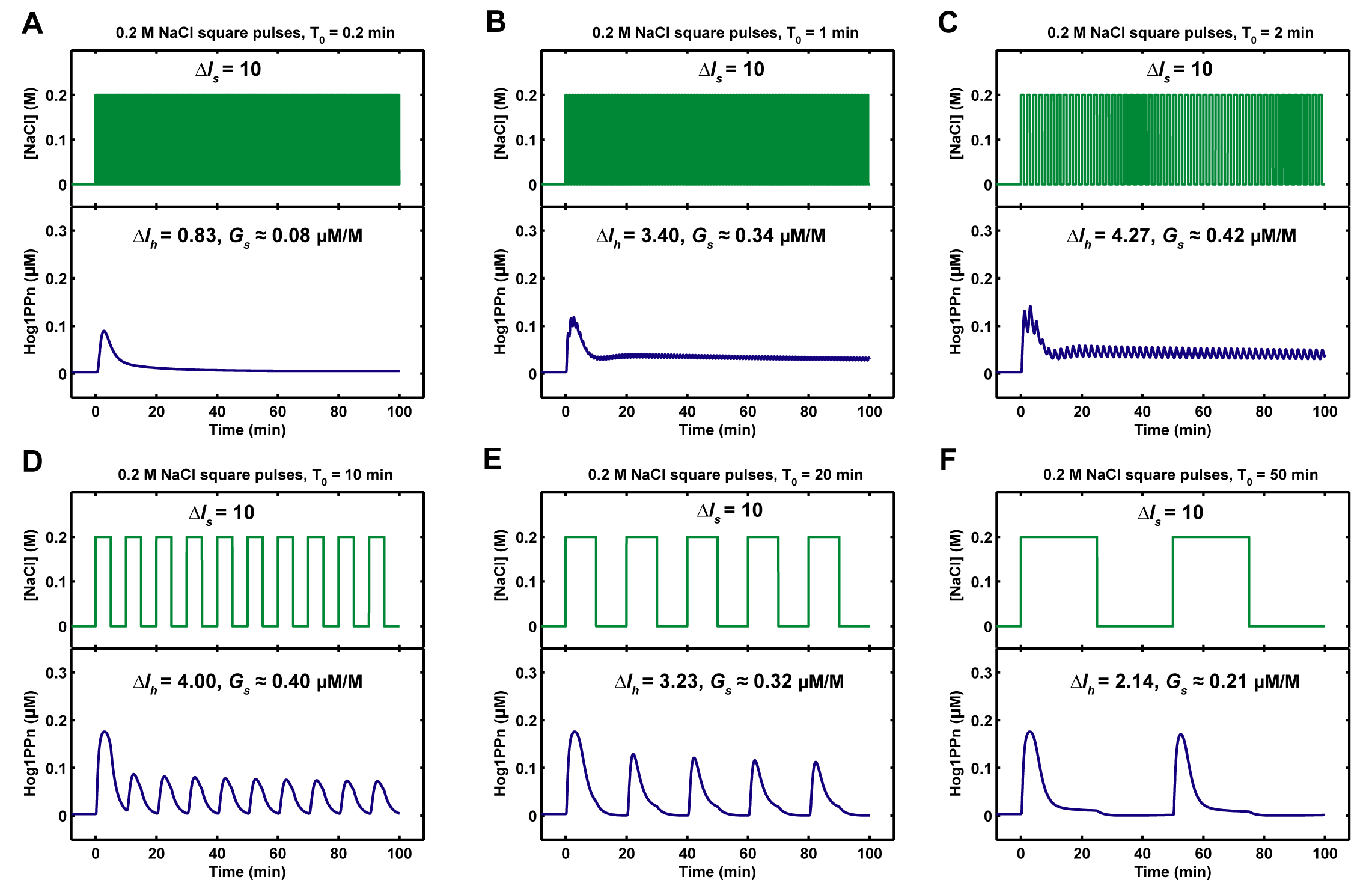

Supplement: Figure S5 — Model predictions for nuclear phosphorylated Hog1 (Hog1PPn) response to different periodic square pulses of 0.2 M NaCl. In all cases, the on time (Ton) and off time (Toff) of square pulses are half of the period time (T0): Ton = Toff = T0/2. ΔIs: NaCl integral change, ΔIh: integral change of Hog1PPn response, Gs: Hog1PPn response gain. (0.50 MB TIF) [file pone.0009522.s005.tif]

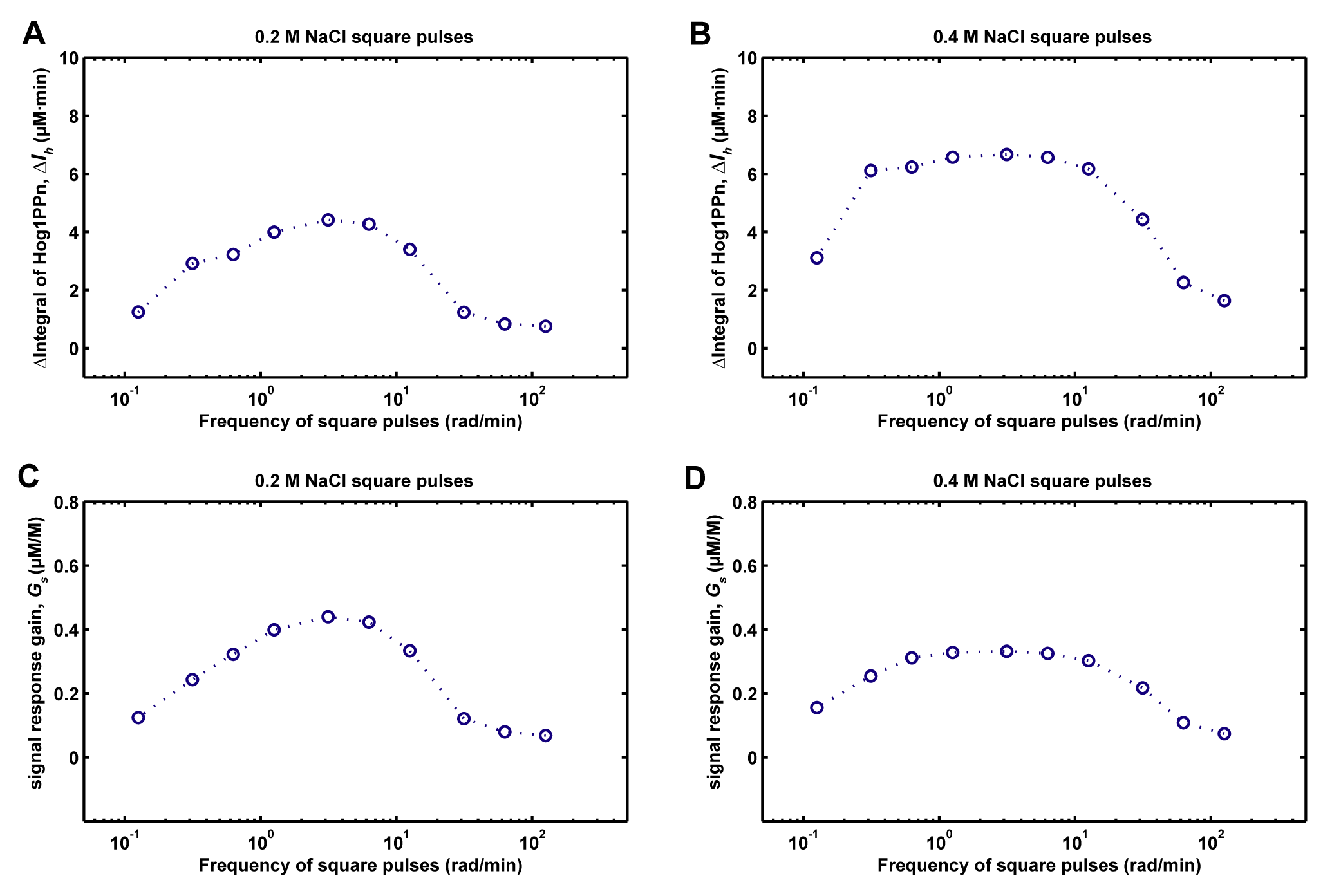

Supplement: Figure S6 — Relationship between nuclear phosphorylated Hog1 (Hog1PPn) response and the duration of periodic square pulses of NaCl change. ΔIh: integral change of Hog1PPn response. Gs: Hog1PPn response gain. (0.33 MB TIF) [file pone.0009522.s006.tif]

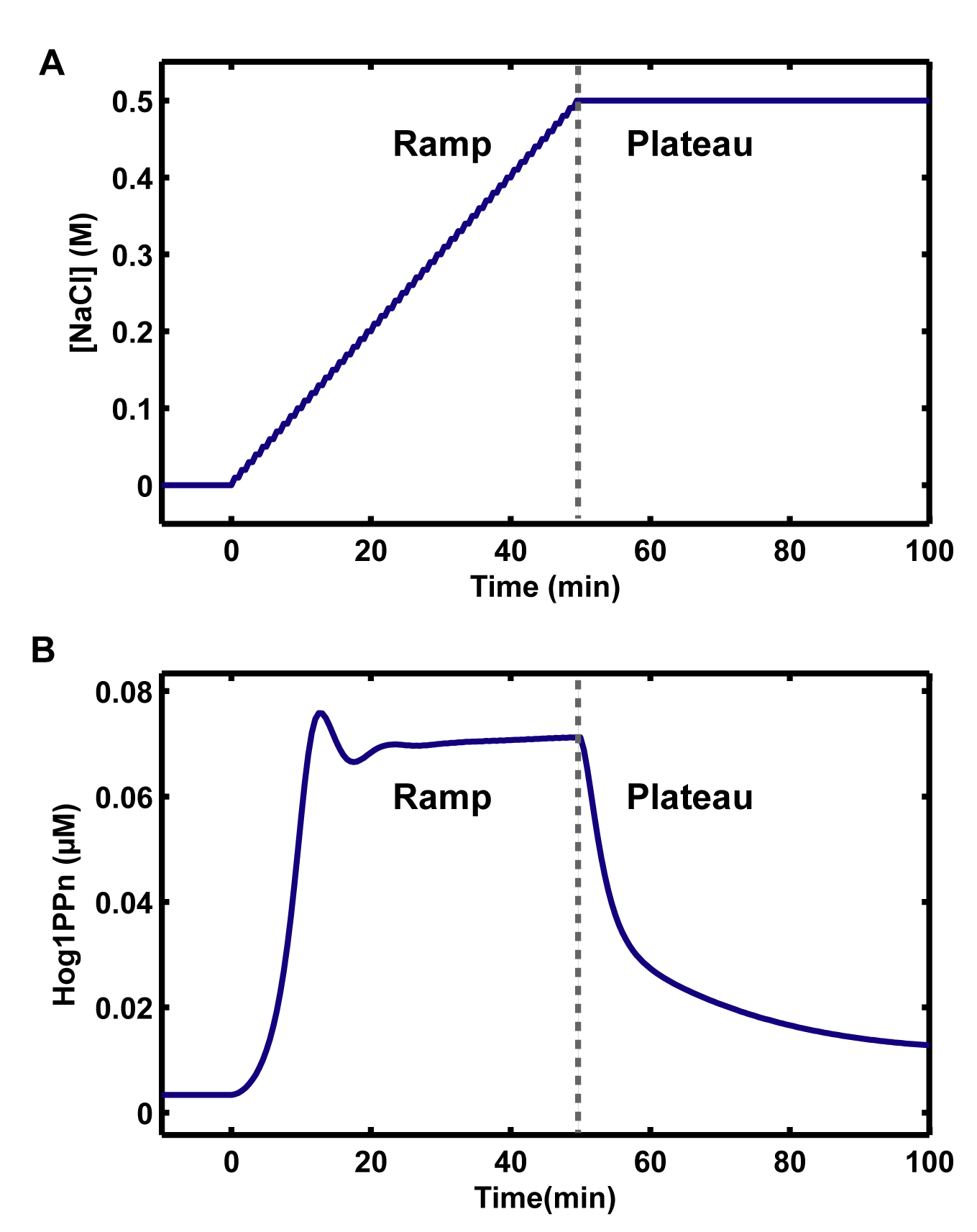

Supplement: Figure S7 — Model prediction for nuclear phosphorylated Hog1 (Hog1PPn) response to ramp increase of NaCl. (0.24 MB TIF) [file pone.0009522.s007.tif]

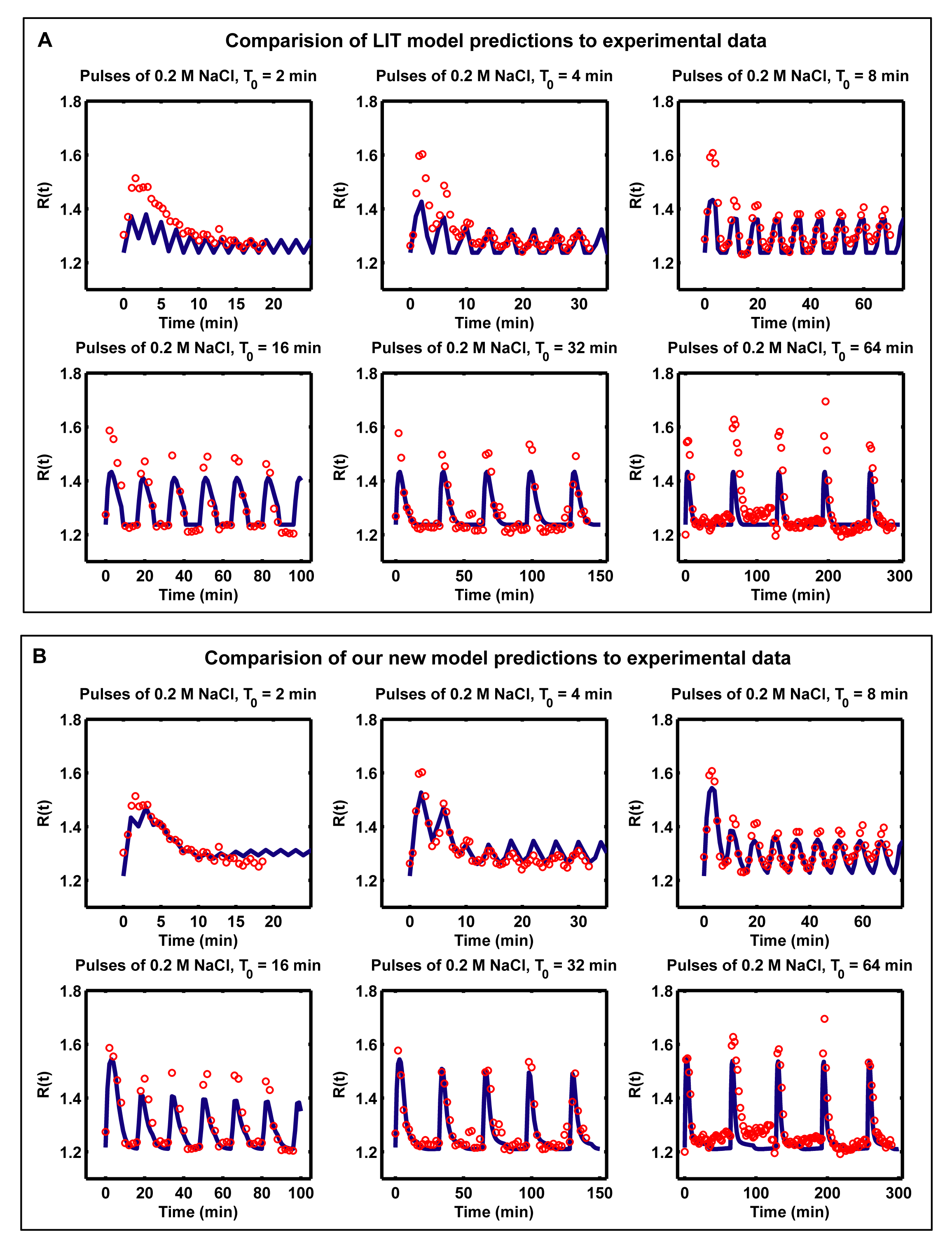

Supplement: Figure S8 — Comparison of the data fitting of the LIT concise model and our new model for periodic square pulses of 0.2 M NaCl. The red circle points are the experimental data sets in Fig. S2 of the reference (Mettetal et al., Science, 2008, 319: 482–484). (1.01 MB TIF) [file pone.0009522.s008.tif]

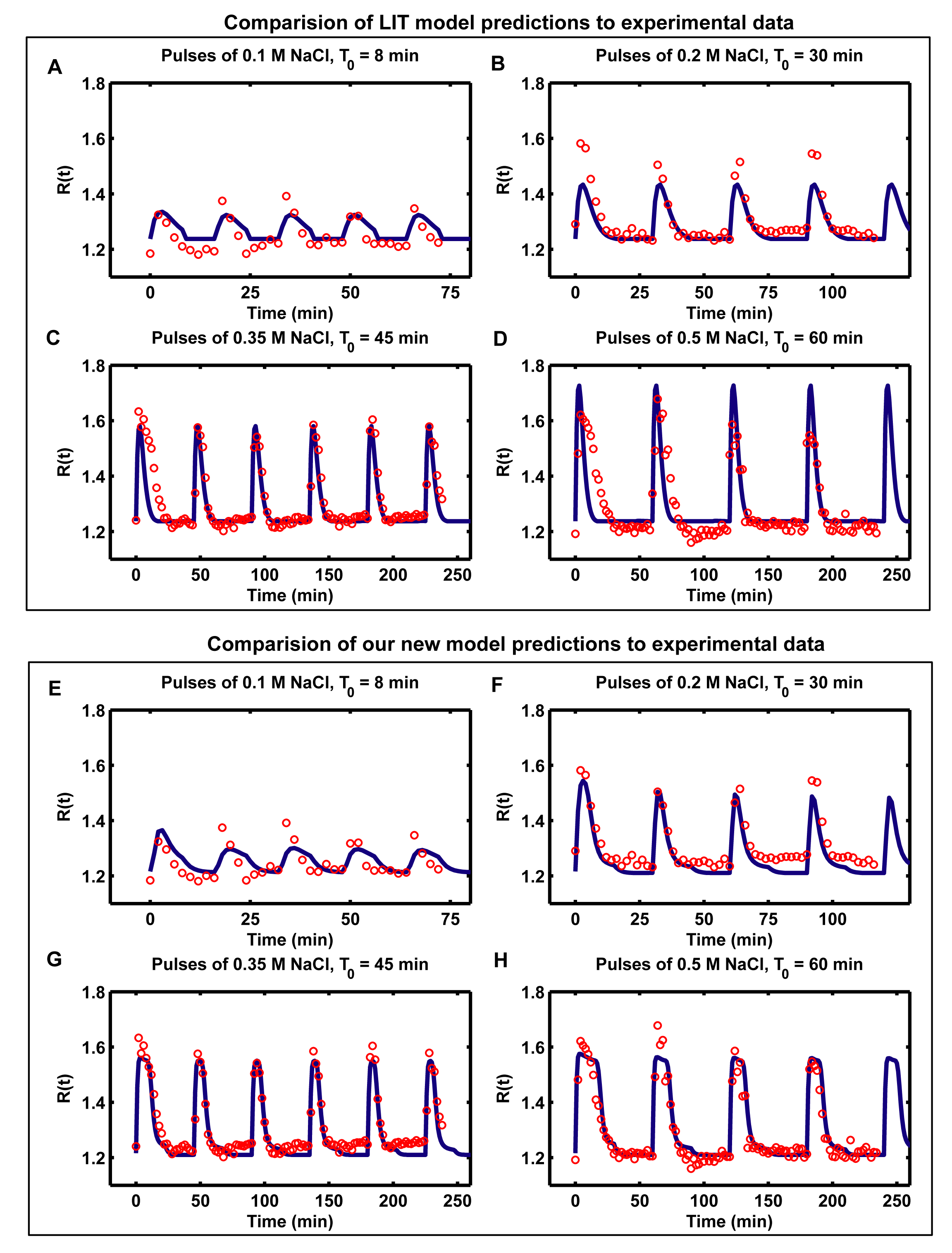

Supplement: Figure S9 — Comparison of the data fitting of the LIT concise model and our new model for different periodic square pulses of NaCl stimulation. The red circle points are the experimental data sets in Fig. S5 of the reference (Mettetal et al., Science, 2008, 319: 482–484). (0.97 MB TIF) [file pone.0009522.s009.tif]

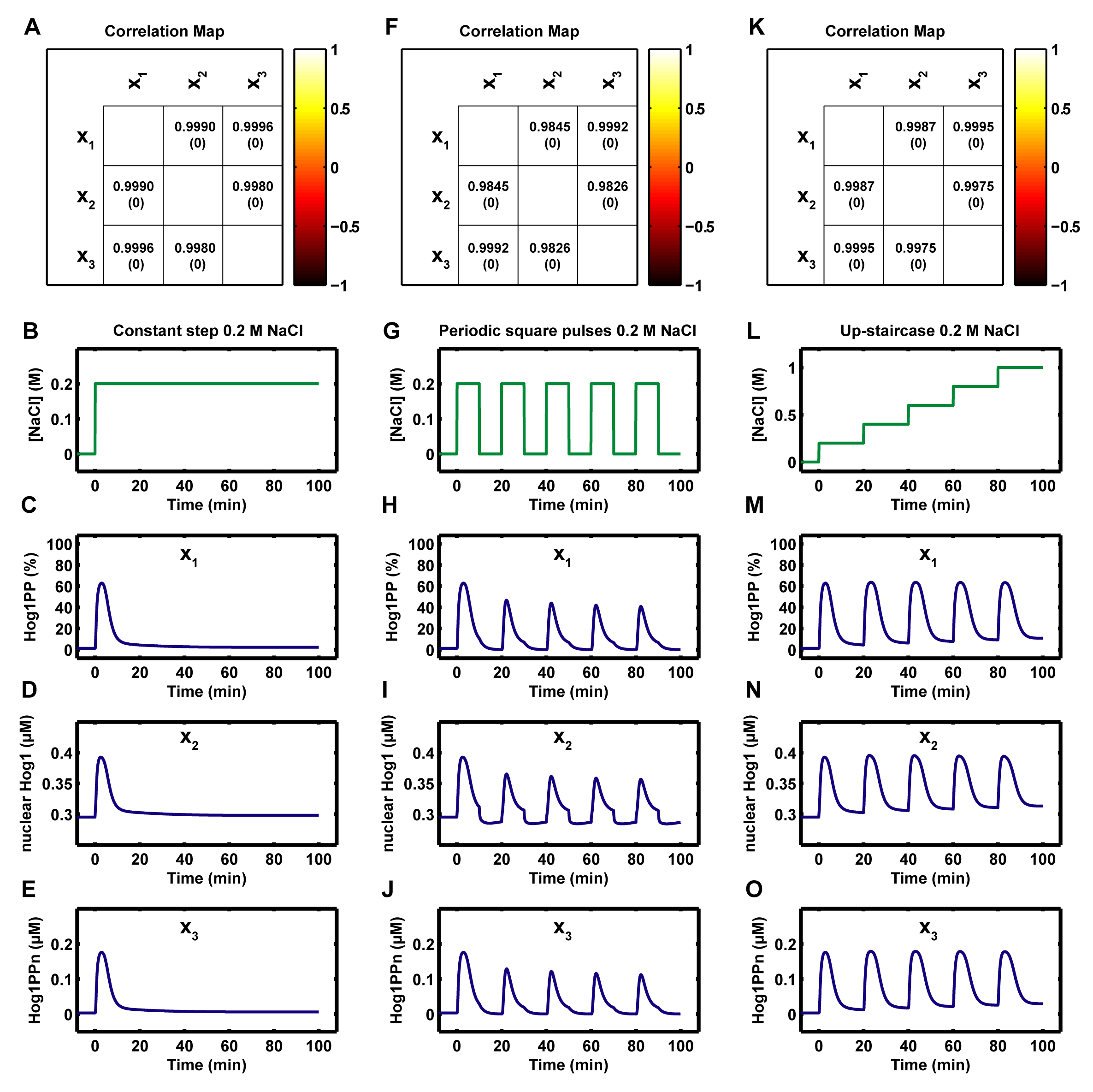

Supplement: Figure S10 — The total amount of phosphorylated Hog1 (x1, Hog1PP, quantified as % of total Hog1), nuclear Hog1 enrichment (x2, nuclear Hog1) and nuclear phosphorylated Hog1 (x3, Hog1PPn) are highly correlated under different types of NaCl stimulation. Numbers in the squares of the correlation map (A, F, K) denote the correlation coefficients of two variables. Numbers within parentheses correspond to the rounded p-values for testing the hypothesis of uncorrelated variables. If the p-value is small (<0.05), then the correlation of the two variables is significant. The correlation coefficient and p-values were calculated with the “corrcoef” function in Matlab. (A–E) Simple step increase of NaCl. (F–J) Periodic square pulses of NaCl. (K–O) Up-staircase increase of NaCl. (0.76 MB TIF) [file pone.0009522.s010.tif]
