# Supplementary material for: A Quantitative Study of the Hog1 MAPK Response to Fluctuating Osmotic Stress in Saccharomyces cerevisiae
Source: PLoS One. 2010 Mar 4;5(3):e9522. doi: 10.1371/journal.pone.0009522 (PMC2831999; doi:10.1371/journal.pone.0009522)
Supplement: Table S1 — Initial conditions of the state variables. (0.05 MB DOC) [file pone.0009522.s011.doc]

**Table S1** Initial conditions of the state variables

| **State variable** | **Initial Condition** | **Annotation** |
| --- | --- | --- |
| *Pbs2* | 0.1231 μM | unphosphorylated Pbs2 |
| *Pbs2PP* | 0.000616 μM | phosphorylated Pbs2 |
| *Hog1c* | 0.3426 μM | unphosphorylated cytoplasmic Hog1 |
| *Hog1PPc* | 0.004443 μM | phosphorylated cytoplasmic Hog1 |
| *Hog1n* | 0.2918 μM | unphosphorylated nuclear Hog1 |
| *Hog1PPn* | 0.00338 μM | phosphorylated nuclear Hog1 |
| *Glyc_in* | 576000 μM | intracellular glycerol |
| *Yt* | 1.811 μM | overall variable for the effect of transcriptional feedback intermediates on glycerol production |
| *Vos* | 34.8 fL (10^-18^ m^3^) | osmotic volume of the cell |
| *z1,* *z2,* *z3,* *z4* | 0.00338 μM | linear chain variables for the delay of transcriptional feedback on glycerol production |

The estimated values for the initial condition of state variables are rounded.
