# Supplementary material for: A Quantitative Study of the Hog1 MAPK Response to Fluctuating Osmotic Stress in Saccharomyces cerevisiae
Source: PLoS One. 2010 Mar 4;5(3):e9522. doi: 10.1371/journal.pone.0009522 (PMC2831999; doi:10.1371/journal.pone.0009522)
Supplement: Table S2 — Complete list of model parameter values. (0.10 MB DOC) [file pone.0009522.s012.doc]

**Table S2. Complete list of model parameter values**

| $K_{pho}^{Pbs2}$ = 39.22 (min^-1^) | α = 0.398 (×10^6^ J∙m^-3^) | $K_{depho}^{Pbs2}$ = 13.44 (min^-1^) |
| --- | --- | --- |
| $K_{pho}^{Hog1}$ = 11.2 (μM^-1^∙min^-1^) | $K_{depho}^{Hog1PPc}$ = 0.09061 (min^-1^) | $K_{imp}^{Hog1c}$ = 0.7528 (min^-1^) |
| $K_{exp}^{Hog1n}$= 6.36 (min^-1^) | $K_{imp}^{Hog1PPc}$= 1.195 (min^-1^) | $K_{exp}^{Hog1PPn}$= 7.076 (min^-1^) |
| $K_{depho}^{Hog1PPn}$= 4.144 (min^-1^) | $K_{s0}^{Glyc}$ = 5248 (μM∙min^-1^) | $K_{s1}^{Glyc}$ = 56140 (μM∙min^-1^) |
| β = 0.2992 (dimensionless) | $K_{s2}^{Glyc}$ = 1139 (min^-1^) | $K_{exp0}^{Glyc}$= 0.005 (min^-1^) |
| $K_{exp1}^{Glyc}$= 0.02963 (min^-1^) | γ = 0.9626 (×10^6^ J∙m^-3^) | $K_{s0}^{Yt}$ = 0.01211 (μM∙min^-1^) |
| $K_{s1}^{Yt}$ = 1.204 (min^-1^) | $K_{t}^{Yt}$ = 0.008934 (min^-1^) | τ = 20 (min) |
| *w* = 4.688 (×10^6^ J∙m^-3^∙M^-1^) | *G* = 72.4607 (×10^-12^ m^2^) | *Lp* = 0.2497 (×10^-12^ m^4^∙J^-1^∙min^-1^) |
| $f^{V_{cyt}}$ = 0.5 (dimensionless) | $f^{V_{nuc}}$ = 0.07 (dimensionless) | $V_{os}^{PI_{t}=0}$ = 30.63 (×10^-18^ m^3^, fL) |
| $V_{cell}^{0}$ = 58 (×10^-18^ m^3^, fL) | $V_{os}^{0}$ = 34.8 (×10^-18^ m^3^, fL) | $V_{b}$ = 23.2 (×10^-18^ m^3^, fL) |
| $PI_{e}^{0}$= 0.625 (×10^6^J∙m^-3^) | $PI_{t}^{0}$= 0.875 (×10^6^ J∙m^-3^) | $PI_{i}^{0}$= 1.5 (×10^6^ J∙m^-3^) |
| n_0_ = 4.176×10^-15^ mol = 4.176×10^6^ (×10^-15^ μmol) (contribution of other osmolarity except glycerol) | | |
| R = 8.314 ([J](http://en.wikipedia.org/wiki/Joule)∙[K](http://en.wikipedia.org/wiki/Kelvin)−1∙[mol](http://en.wikipedia.org/wiki/Mole_(unit))−1) T = 303.15 (K) | | |

The estimated parameter values are rounded. The units of the parameters for osmotic pressure and volume come with some scaling factors for the convenience of numerical simulations.
