# Supplementary material for: A Quantitative Study of the Hog1 MAPK Response to Fluctuating Osmotic Stress in Saccharomyces cerevisiae
Source: PLoS One. 2010 Mar 4;5(3):e9522. doi: 10.1371/journal.pone.0009522 (PMC2831999; doi:10.1371/journal.pone.0009522)
Supplement: Table S3 — Complete list of ordinary differential equations and other equations. (0.10 MB DOC) [file pone.0009522.s013.doc]

**Table S3. Complete list of ordinary differential equations and other equations**

|  | $\frac{d[Pbs2]}{dt}=-\frac{K_{pho}^{Pbs2}\cdot[Pbs2]}{1+\left(\frac{PI_{t}}{\alpha}\right)^{8}}+K_{depho}^{Pbs2}\cdot[Pbs2PP]-[Pbs2]\cdot V_{ratio}$ | (1) |
| --- | --- | --- |
|  | $\frac{d[Pbs2PP]}{dt}=\frac{K_{pho}^{Pbs2}\cdot[Pbs2]}{1+\left(\frac{PI_{t}}{\alpha}\right)^{8}}-K_{depho}^{Pbs2}\cdot[Pbs2PP]-[Pbs2PP]\cdot V_{ratio}$ | (2) |
|  | $\begin{matrix} \frac{d[Hog1c]}{dt}=-K_{pho}^{Hog1}\cdot[Pbs2PP]\cdot[Hog1c]+K_{depho}^{Hog1PPc}\cdot[Hog1PPc] \\ \text{ }-K_{imp}^{Hog1c}\cdot[Hog1c]+\frac{K_{exp}^{Hog1n}\cdot[Hog1n]\cdot V_{nuc}}{V_{cyt}}-[Hog1c]\cdot V_{ratio} \end{matrix}$ | (3) |
|  | $\begin{matrix} \frac{d[Hog1PPc]}{dt}=K_{pho}^{Hog1}\cdot[Pbs2PP]\cdot[Hog1c]-K_{depho}^{Hog1PPc}\cdot[Hog1PPc] \\ \text{ }-K_{imp}^{Hog1PPc}\cdot[Hog1PPc]+\frac{K_{exp}^{Hog1PPn}\cdot[Hog1PPn]\cdot V_{nuc}}{V_{cyt}} \\ \text{ }-[Hog1PPc]\cdot V_{ratio} \end{matrix}$ | (4) |
|  | $\begin{matrix} \frac{d[Hog1n]}{dt}=\frac{K_{imp}^{Hog1c}\cdot[Hog1c]\cdot V_{cyt}}{V_{nuc}}-K_{exp}^{Hog1n}\cdot[Hog1n]+K_{depho}^{Hog1PPn}\cdot[Hog1PPn] \\ \text{ }-[Hog1n]\cdot V_{ratio} \end{matrix}$ | (5) |
|  | $\begin{matrix} \frac{d[Hog1PPn]}{dt}=\frac{K_{imp}^{Hog1PPc}\cdot[Hog1PPc]\cdot V_{cyt}}{V_{nuc}}-K_{exp}^{Hog1PPn}\cdot[Hog1PPn] \\ \text{ }-K_{depho}^{Hog1PPn}\cdot[Hog1PPn]-[Hog1PPn]\cdot V_{ratio} \end{matrix}$ | (6) |
|  | $\begin{matrix} \frac{d[Glyc\_in]}{dt}=K_{s0}^{Glyc}+\frac{K_{s1}^{Glyc}\cdot(totalHog1PP)^{4}}{\beta^{4}+(totalHog1PP)^{4}}+K_{s2}^{Glyc}\cdot[Yt] \\ \text{ }-\left(K_{exp0}^{Glyc}+\frac{K_{exp1}^{Glyc}\cdot(PI_{t})^{12}}{(\gamma)^{12}+(PI_{t})^{12}}\right)\cdot([Glyc\_in]-[Glyc\_ex]) \\ \text{ }-[Glyc\_in]\cdot V_{ratio} \end{matrix}$ | (7) |
|  | $\frac{d[Yt]}{dt}=K_{s0}^{Yt}+K_{s1}^{Yt}\cdot[z4]-K_{t}^{Yt}\cdot[Yt]-[Yt]\cdot V_{ratio}$ | (8) |

**Table S3 (Continued)**

|  | $\frac{d[z1]}{dt}=\frac{4\cdot([Hog1PPn]-[z1])}{\tau}$ | (9) |
| --- | --- | --- |
|  | $\frac{d[z2]}{dt}=\frac{4\cdot([z1]-[z2])}{\tau}$ | (10) |
|  | $\frac{d[z3]}{dt}=\frac{4\cdot([z2]-[z3])}{\tau}$ | (11) |
|  | $\frac{d[z4]}{dt}=\frac{4\cdot([z3]-[z4])}{\tau}$ | (12) |
|  | $\frac{d(V_{os})}{dt}=-G\cdot Lp\cdot(PI_{e}+PI_{t}-PI_{i})$ | (13) |
|  | $PI_{e}=PI_{e}^{0}+w\cdot[NaCl]$ | (14) |
|  | $PI_{t}=\left\{\begin{matrix} \frac{PI_{t}^{0}\cdot(V_{os}-V_{os}^{PI_{t}=0})}{(V_{os}^{0}-V_{os}^{PI_{t}=0})}\text{ if }V_{os}^{0}\text{ }>V_{os}^{PI_{t}=0} \\ 0\text{ if }V_{os}^{0}\text{ }\leq V_{os}^{PI_{t}=0} \end{matrix}\right.$ | (15) |
|  | $V_{cell}=V_{os}+V_{b}$ | (16) |
|  | $V_{cyt}=f^{V_{cyt}}\cdot V_{cell}=0.5\cdot V_{cell}$ | (17) |
|  | $V_{nuc}=f^{V_{nuc}}\cdot V_{cell}=0.07\cdot V_{cell}$ | (18) |
|  | $\begin{matrix} PI_{i}=\frac{n\cdot R\cdot T}{V_{os}}=\frac{n_{0}+n_{Glyc}}{V_{os}}\cdot R\cdot T=\frac{n_{0}+[Glyc\_in]\cdot V_{cyt}}{V_{os}}\cdot R\cdot T \\ \text{ }=\frac{n_{0}+[Glyc\_in]\cdot V_{cyt}}{V_{os}}\cdot10^{-9}\cdot8.314\cdot303.15 \end{matrix}$ | (19) |
|  | $totalHog1PP=\frac{([Hog1PPc]\cdot V_{cyt}+[Hog1PPn]\cdot V_{nuc})\cdot602}{6780}$ | (20) |

**Table S3 (Continued)**

|  | $\begin{matrix} V_{ratio}=\frac{1}{V_{cell}}\cdot\frac{d(V_{cell})}{dt}=\frac{1}{V_{cell}}\cdot\frac{d(V_{os}+V_{b})}{dt}=\frac{1}{V_{cell}}\cdot\frac{d(\cdot V_{os})}{dt} \\ \text{ }=\frac{-G\cdot Lp\cdot(PI_{e}+PI_{t}-PI_{i})}{V_{cell}} \end{matrix}$ | (21) |
| --- | --- | --- |
|  | $\begin{matrix} Rt=\frac{\text{ }[Hog1n]+[Hog1PPn]\text{ }}{\frac{\left(\frac{N_{Hog1}^{total}}{N_{a}}\right)}{(V_{cell}-V_{cell}^{wall})}} \\ \text{ }=\frac{([Hog1n]+[Hog1PPn])\cdot0.8\cdot(Vos+Vb)\cdot602}{6780} \end{matrix}$ (note: [*Hog*1*n*] and [*Hog*1*PPn*] have unit of 10^-6^ M; *V_cell_* has unit of 10^-15^ L) | (22) |
